# Mitochondrial dysfunction and mitophagy defects in *LRRK2-R1441C* Parkinson’s disease models

**DOI:** 10.1093/hmg/ddad102

**Published:** 2023-06-29

**Authors:** Matthew G Williamson, Marta Madureira, William McGuinness, Rachel Heon-Roberts, Elliot D Mock, Kalina Naidoo, Kaitlyn M L Cramb, Maria-Claudia Caiazza, Ana B Malpartida, Martha Lavelle, Katrina Savory, Stewart W Humble, Ryan Patterson, John B Davis, Natalie Connor-Robson, Brent J Ryan, Richard Wade-Martins

**Affiliations:** Oxford Parkinson’s Disease Centre and Department of Physiology, Anatomy and Genetics, University of Oxford, Oxford OX1 3QU, UK; Kavli Institute for Nanoscience Discovery, Dorothy Crowfoot Hodgkin Building, University of Oxford, Oxford OX1 3QU, UK; Oxford Parkinson’s Disease Centre and Department of Physiology, Anatomy and Genetics, University of Oxford, Oxford OX1 3QU, UK; Kavli Institute for Nanoscience Discovery, Dorothy Crowfoot Hodgkin Building, University of Oxford, Oxford OX1 3QU, UK; ICBAS, Instituto de Ciências Biomédicas de Abel Salazar, Universidade do Porto, Rua Jorge de Viterbo Ferreira, 228, Porto 4050-313, Portugal; Oxford Parkinson’s Disease Centre and Department of Physiology, Anatomy and Genetics, University of Oxford, Oxford OX1 3QU, UK; Kavli Institute for Nanoscience Discovery, Dorothy Crowfoot Hodgkin Building, University of Oxford, Oxford OX1 3QU, UK; Oxford Parkinson’s Disease Centre and Department of Physiology, Anatomy and Genetics, University of Oxford, Oxford OX1 3QU, UK; Kavli Institute for Nanoscience Discovery, Dorothy Crowfoot Hodgkin Building, University of Oxford, Oxford OX1 3QU, UK; Oxford Parkinson’s Disease Centre and Department of Physiology, Anatomy and Genetics, University of Oxford, Oxford OX1 3QU, UK; Kavli Institute for Nanoscience Discovery, Dorothy Crowfoot Hodgkin Building, University of Oxford, Oxford OX1 3QU, UK; Oxford Parkinson’s Disease Centre and Department of Physiology, Anatomy and Genetics, University of Oxford, Oxford OX1 3QU, UK; Kavli Institute for Nanoscience Discovery, Dorothy Crowfoot Hodgkin Building, University of Oxford, Oxford OX1 3QU, UK; Oxford Parkinson’s Disease Centre and Department of Physiology, Anatomy and Genetics, University of Oxford, Oxford OX1 3QU, UK; Kavli Institute for Nanoscience Discovery, Dorothy Crowfoot Hodgkin Building, University of Oxford, Oxford OX1 3QU, UK; Oxford Parkinson’s Disease Centre and Department of Physiology, Anatomy and Genetics, University of Oxford, Oxford OX1 3QU, UK; Kavli Institute for Nanoscience Discovery, Dorothy Crowfoot Hodgkin Building, University of Oxford, Oxford OX1 3QU, UK; Oxford Parkinson’s Disease Centre and Department of Physiology, Anatomy and Genetics, University of Oxford, Oxford OX1 3QU, UK; Oxford Parkinson’s Disease Centre and Department of Physiology, Anatomy and Genetics, University of Oxford, Oxford OX1 3QU, UK; Kavli Institute for Nanoscience Discovery, Dorothy Crowfoot Hodgkin Building, University of Oxford, Oxford OX1 3QU, UK; Oxford Parkinson’s Disease Centre and Department of Physiology, Anatomy and Genetics, University of Oxford, Oxford OX1 3QU, UK; Kavli Institute for Nanoscience Discovery, Dorothy Crowfoot Hodgkin Building, University of Oxford, Oxford OX1 3QU, UK; Oxford Parkinson’s Disease Centre and Department of Physiology, Anatomy and Genetics, University of Oxford, Oxford OX1 3QU, UK; Kavli Institute for Nanoscience Discovery, Dorothy Crowfoot Hodgkin Building, University of Oxford, Oxford OX1 3QU, UK; National Institute of Neurological Disorders and Stroke, National Institutes of Health , Bethesda, MD, 20892, USA; Oxford Parkinson’s Disease Centre and Department of Physiology, Anatomy and Genetics, University of Oxford, Oxford OX1 3QU, UK; National Institute of Neurological Disorders and Stroke, National Institutes of Health , Bethesda, MD, 20892, USA; Oxford Drug Discovery Institute, Centre of Medicines Discovery, University of Oxford, NDM Research Building, Old Road Campus, Oxford OX3 7FZ, UK; Oxford Parkinson’s Disease Centre and Department of Physiology, Anatomy and Genetics, University of Oxford, Oxford OX1 3QU, UK; Oxford Parkinson’s Disease Centre and Department of Physiology, Anatomy and Genetics, University of Oxford, Oxford OX1 3QU, UK; Kavli Institute for Nanoscience Discovery, Dorothy Crowfoot Hodgkin Building, University of Oxford, Oxford OX1 3QU, UK; Oxford Parkinson’s Disease Centre and Department of Physiology, Anatomy and Genetics, University of Oxford, Oxford OX1 3QU, UK; Kavli Institute for Nanoscience Discovery, Dorothy Crowfoot Hodgkin Building, University of Oxford, Oxford OX1 3QU, UK

## Abstract

Mutations in the Leucine-Rich Repeat Kinase 2 (*LRRK2*) gene have been identified as one of the most common genetic causes of Parkinson’s disease (PD). The *LRRK2* PD-associated mutations *LRRK2*^*G2019S*^ and *LRRK2*^*R1441C*^, located in the kinase domain and in the ROC-COR domain, respectively, have been demonstrated to impair mitochondrial function. Here, we sought to further our understanding of mitochondrial health and mitophagy by integrating data from *LRRK2*^*R1441C*^ rat primary cortical and human induced pluripotent stem cell-derived dopamine (iPSC-DA) neuronal cultures as models of PD. We found that *LRRK2*^*R1441C*^ neurons exhibit decreased mitochondrial membrane potential, impaired mitochondrial function and decreased basal mitophagy levels. Mitochondrial morphology was altered in *LRRK2*^*R1441C*^ iPSC-DA but not in cortical neuronal cultures or aged striatal tissue, indicating a cell-type-specific phenotype. Additionally, *LRRK2*^*R1441C*^ but not *LRRK2*^*G2019S*^ neurons demonstrated decreased levels of the mitophagy marker pS65Ub in response to mitochondrial damage, which could disrupt degradation of damaged mitochondria. This impaired mitophagy activation and mitochondrial function were not corrected by the LRRK2 inhibitor MLi-2 in *LRRK2*^*R1441C*^ iPSC-DA neuronal cultures. Furthermore, we demonstrate LRRK2 interaction with MIRO1, a protein necessary to stabilize and to anchor mitochondria for transport, occurs at mitochondria, in a genotype-independent manner. Despite this, we found that degradation of MIRO1 was impaired in *LRRK2*^*R1441C*^ cultures upon induced mitochondrial damage, suggesting a divergent mechanism from the *LRRK2*^*G2019S*^ mutation.

## Graphical Abstract

**Graphical Abstract:**
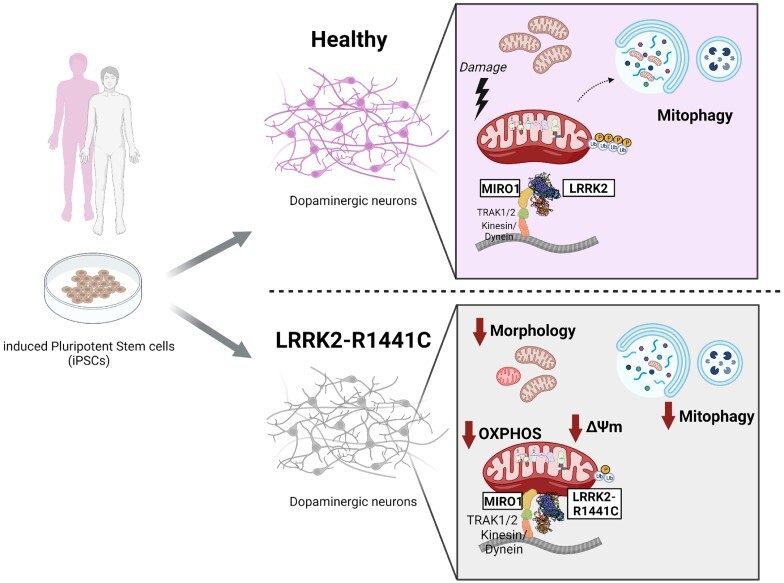

## Introduction

Parkinson’s disease (PD) is the second most common neurodegenerative disorder worldwide, affecting 2% of individuals over 60 years old. PD neuropathology is characterized by progressive loss of dopaminergic neurons in the *substantia nigra pars compacta* (*SNpc*) and an accumulation of alpha-synuclein-positive aggregates in Lewy bodies (1). Mitochondrial dysfunction has long been observed in sporadic PD (2–4). Additionally, mutations in both autosomal dominant and recessive PD-causing genes, including Leucine-Rich Repeat Kinase 2 (*LRRK2*), PTEN-induced kinase *1* (*PINK1*) and Parkin (*PRKN*), have been associated with mitochondrial dysfunction (5–7).

LRRK2 is a large multi-functional protein containing seven functional domains, with both GTPase and kinase activity. The two most common mutations found in PD patients are G2019S in the kinase domain, which shows increased kinase activity, and R1441C in the GTPase domain decreasing GTPase activity (8–10). Conflicting evidence suggests that the *LRRK2^R1441C^* mutation increases kinase activity (10), yet other reports suggest that it does not directly enhance kinase activity (9,11,12).

LRRK2 plays important functional roles within cells, operating as a scaffolding unit in signaling pathways (13), phosphorylates several substrates of the Rab family of proteins to regulate endosomal and vesicle trafficking (12,14,15) and is involved in cytoskeleton dynamics and neurite outgrowth by interacting with microtubules (16,17). Mutations in LRRK2 have been shown to modulate autophagy/mitophagy and mitochondrial function, processes highly implicated in the pathology of PD (15,18–20). Our previous work demonstrated that primary cortical neuronal cultures of BAC transgenic rats expressing the *LRRK2^R1441C^* mutation exhibit decreased autophagy-lysosomal fusion, altered lysosomal pH and decreased lysosomal protein degradation, deficits not found in *LRRK2^WT^* or *LRRK2^G2019S^* transgenic neurons (21). Although *LRRK2^R1441C^* cortical primary neuronal cultures displayed autophagy defects, mitochondrial health and mitophagy were not characterized in these models.

Several studies have shown decreased mitochondrial membrane potential, reduced rate of OXPHOS, decreased ATP production, aberrant mitochondrial morphology, mitochondrial fragmentation and decreased mitophagy in *LRRK2^G2019S^* fibroblasts (22–27). Basal mitophagy inversely correlates with LRRK2 kinase activity *in vivo* and the *LRRK2^G2019S^* mutation impairs basal mitophagy in reporter mouse models (28). Recently, a study demonstrated altered mitochondrial morphology and dysfunction with decreased mitophagy in *LRRK2^R1441G^* transgenic mice (29). The *LRRK2^R1441C^* mutation also impairs depolarization-induced mitophagy and mitochondrial function in primary human skin fibroblasts (27).

Recently, independent studies have identified three main mechanisms linking LRRK2 to regulation of mitophagy. In human induced pluripotent stem cell-derived dopaminergic (iPSC-DA) neurons, the *LRRK2^G2019S^* mutation decreased interaction with outer mitochondrial membrane (OMM) protein MIRO1 (*RHOT1*), which leads to decreased MIRO1 degradation in depolarized mitochondria and consequent decrease in mitophagy levels (30). Also in depolarized mitochondria, the *LRRK2^G2019S^* and *LRRK2^R1441C^* mutations block PINK1/Parkin-dependent mitophagy through increased phosphorylation of the LRRK2 substrate Rab10, which inhibits its interaction with the autophagy receptor optineurin in primary fibroblasts (27). In parallel, the *LRRK2^G2019S^* mutation interferes with protein–protein interactions involving Parkin and fission protein Drp1 on the OMM, resulting in decreased mitophagy in human primary fibroblasts (22). However, the effect of the *LRRK2^R1441C^* mutation on mitochondrial network, mitochondrial function and mitophagic clearance in dopaminergic neurons, the neuronal subtype most affected in PD, is still poorly understood.

In the present study, we investigated mitochondrial health and mitophagy under both basal and stress conditions using *in vitro* LRRK2 models of Parkinson’s disease. We focused on the *LRRK2^R1441C^* mutation using rat primary cortical neuronal and iPSC-DA neuronal models and observe altered mitochondrial morphology and function in addition to impaired depolarization-induced pS65Ub levels and decrease in mitochondria clearance. We report that this mitochondrial dysfunction could not be rescued in *LRRK2^R1441C^* iPSC-DA neuronal cultures by LRRK2 kinase inhibition. Finally, we confirmed that impaired MIRO1 degradation underlies the mitochondrial dysfunction in *LRRK2^R1441C^* iPSC-DA neuronal cultures.

## Results

### Primary rat *LRRK2^R1441C^* cortical neuronal cultures exhibit mitochondrial damage and impaired mitophagy

**Figure 1:**
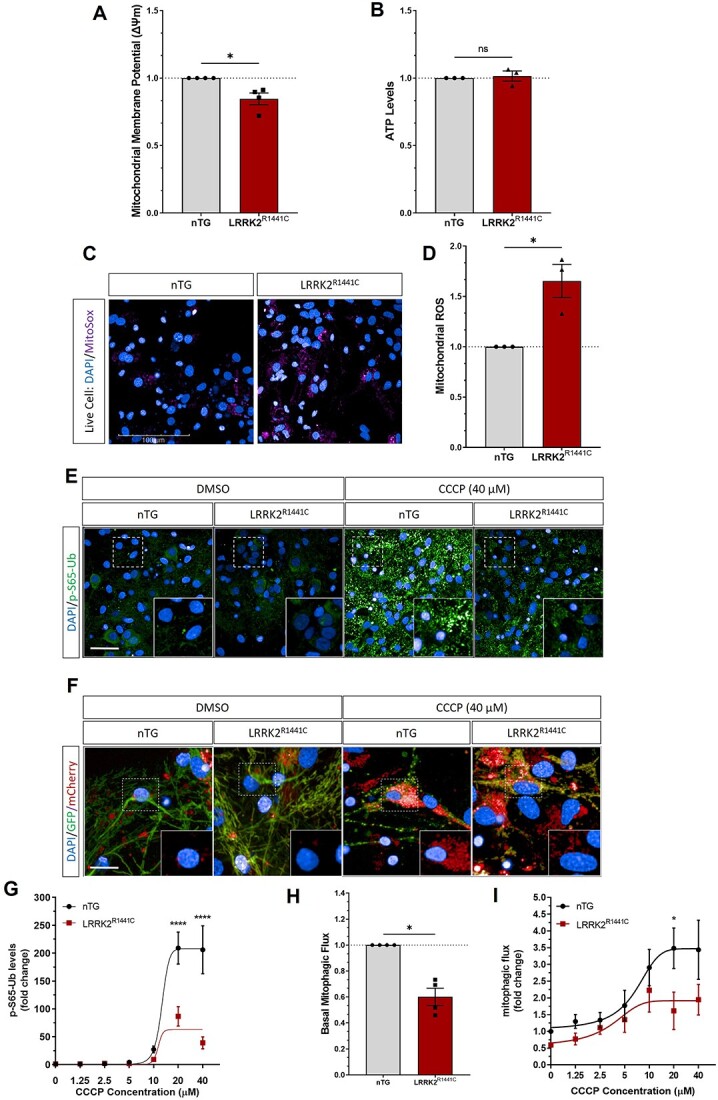
Mitochondrial dysfunction and reduced mitophagy levels in primary cortical neuronal cultures from *LRRK2^R1441C^* transgenic rats. Primary cortical neuronal cultures were generated from P1 to P5 pups and assayed at DIV 14 for (**A**) mitochondrial membrane potential measured using JC-10 dye, (**B**) ATP levels measured using a luminescent assay and (**C**, **D**) mitochondrial ROS production using MitoSox changes measured in *LRRK2^R1441C^* cortical cultures, normalized to non-transgenic littermate levels. Data points represent averaged transgenic genotypes. Bars, mean ± SEM from three independent litters (ns: non-significant; ^*^P < 0.05; Mann–Whitney unpaired *t* test). (**E**, **G**) DIV 14 primary cortical cultures were treated CCCP for 8 h and pS65Ub was quantified in MAP2 and expressed as number of pS65Ub puncta per neuronal area was quantified. (**F**) DIV 14 primary cortical cultures were transduced with Mito-QC lentivirus reporter, treated with CCCP for 8 h. Mitophagic flux was assessed by quantifying mCherry puncta intensity and number in transfected cells under basal conditions (**H**) or after CCCP treatment (**I**). Data points represent averaged litters per each genotype. Error bars: mean ± SEM from at least three independent litters (ns: non-significant; ^*^P < 0.05; ^*^^*^P < 0.01; ^*^^*^^*^P < 0.001; ^*^^*^^*^^*^P < 0.0001; unpaired *t* test).

To investigate the role of PD-associated *LRRK2* mutations on mitochondrial structure and function, we generated primary cortical neuronal cultures from *LRRK2* BAC transgenic rat pups (P1–P5) expressing the human *LRRK2^hWT^*, *LRRK2^G2019S^* or *LRRK2^R1441C^* transgene, and non-transgenic (nTG) littermates (21,31). First, we measured mitochondrial membrane potential (ΔΨm) using the potentiometric JC-10 dye. Under basal conditions, *LRRK2*^*R1441*C^ cortical neuronal cultures show a significant decrease in ΔΨm, compared with nTG littermates (*P* = 0.012, Mann–Whitney *t* test) (Fig. 1A). These changes in *LRRK2^R1441C^* cultures were coupled with a significant, >1.5-fold, increase in mitochondrial ROS production, as assessed using MitoSox dye (*P* = 0.0166) (Fig. 1C and D), but were independent of changes in cellular ATP levels and alterations in mitochondrial morphology (Fig. 1B; Supplementary Material, Fig. S1A–C). In parallel, we also assessed mitochondrial function in *LRRK2^hWT^* and *LRRK2^G2019S^* primary cortical neuronal cultures and observed no differences in mitochondrial membrane potential, ATP levels, mitochondrial ROS or basal mitophagy flux, in *LRRK2^hWT^* and *LRRK2^G2019S^* compared with nTG littermates (Supplementary Material, Fig. S1D–L).

Following these findings of impaired mitochondrial health in *LRRK2^R1441C^* primary neurons, we assessed if clearance of damaged mitochondria through mitophagy was altered in neuronal cultures. Given mitophagy levels have been reported to be altered under both basal and depolarized conditions in *LRRK2* models, we developed an immunofluorescence staining of phosphorylated ubiquitin (pS65Ub), a marker of PINK1 activation and mitophagy initiation. Specificity of pS65Ub induction was confirmed using stable SH-SY5Y cell lines with a CRISPR/Cas9-mediated knockout of *PINK1*. In contrast to WT cells, cells lacking PINK1 demonstrated no induction of pS65Ub after CCCP treatment (10 μm; 6 h) (Supplementary Material, Fig. S2). In addition, the mitophagy reporter *Mito-QC* was used to assess mitophagy in primary neuronal cortical cultures (Fig. 1E and F). CCCP-induced pS65Ub puncta number was significantly decreased in *LRRK2^R1441C^* cells when compared with nTG cultures (*P* ≤ 0.0001, unpaired *t* test) (Fig. 1E and G). To confirm that decreased pS65Ub induction resulted in impaired mitophagy, we assessed mitophagic flux in *LRRK2* primary cortical neuronal cultures transduced with *Mito-QC* reporter lentivirus (32,33). We observed a 50% decrease in mitophagy in *LRRK2^R1441C^* neuronal cultures, as assessed by the number of mito-lysosomes (red puncta per cell) under basal conditions (*P* = 0.0286) (Fig. 1F and H). Additionally, depolarization-induced mitophagy in *LRRK2^R1441C^* primary neuronal cultures was also decreased compared with nTG (*P* = 0.0409, two-way ANOVA) (Fig. 1F and I). Together, these data demonstrate impairments in mitochondrial function and PINK1-dependent (depolarization-induced) mitophagy in *LRRK2^R1441C^* primary cortical cultures.

The dopaminergic neurons of the *SNpc* that are affected in PD project to the dorsal striatum. We therefore investigated mitochondrial morphology in dorsal striatal brain tissue from aged (21–22 months old) *LRRK2* BAC transgenic rats and nTG littermates. Striatal tissue sections were imaged using electron microscopy to analyze mitochondrial morphology (Supplementary Material, Fig. S3A). Mitochondrial area and mitochondrial roundness were calculated and compared between animals expressing *LRRK2* wild-type and mutant proteins. We observed that mitochondrial area was not changed in *LRRK2^R1441C^* and *LRRK2^G2019S^* aged striatum (*P* = 0.2683 and 0.3388, respectively) (Supplementary Material, Fig. S3B). However, mitochondrial roundness was altered in *LRRK2^G2019S^* animals when compared with non-transgenic littermates (*P* = 0.0197), but not altered in *LRRK2^R1441C^* and *LRRK2^hWT^* (Supplementary Material, Fig. S3C).

Taken together, these data suggest that mitochondrial health is impaired in *LRRK2^R1441C^* cortical neuronal cultures but not in *LRRK2^hWT^* and *LRRK2^G2019S^* cells *in vitro*. Striatal tissue sections from aged *LRRK2^G2019S^* rats exhibited rounder mitochondria, which could indicate a perturbed mitochondrial network, although no change in mitochondrial function was seen in *LRRK2^G2019S^* primary cortical neurons *in vitro*.

### *LRRK2^R1441C^* iPSC-DA neuronal cultures show mitochondrial alterations and dysfunction

Given the mitochondrial dysfunction and impaired mitophagy observed in *LRRK2^R1441C^* rat cortical neuronal cultures, we assessed if this dysfunction was present in iPSC-derived dopamine (DA) neuronal cultures from Parkinson’s patients with *LRRK2^R1441C^* mutations, a highly physiological model of PD. iPSCs derived from three patients with PD carrying the *LRRK2^R1441C^* mutation, and three healthy controls, were differentiated to midbrain dopaminergic neurons. The presence of mature neurons (MAP2^+^) and the percentage of tyrosine hydroxylase-positive (TH^+^) dopamine neurons was consistent across genotypes and multiple differentiations (Supplementary Material, Fig. S4). Endogenous LRRK2 expression and LRRK2-mediated phosphorylation of Rab substrates were confirmed by western blotting in control iPSC-DA neuronal cultures treated with LRRK2 kinase inhibitor MLi-2, using an iPSC-DA neuronal culture derived from a previously described *LRRK2-Knockout* (*LRRK2-KO*) iPSC line for validation (34) (Supplementary Material, Fig. S5A–C). Endogenous LRRK2 expression levels were similar between *LRRK2^R1441C^* and control iPSC-DA neuronal cultures, as assessed by western blotting (*P* = 0.7686; Supplementary Material, Fig. S5D and E). It is important to note that owing to high sequence homology, the phosphorylated Rab8A (T72) antibody commercially available also cross-reacts with phosphorylated Rab3A, Rab10, Rab35 and Rab43 and as such resulting detection will be referred to as phospho-Rabs (35). Thus, change in phosphorylation of LRRK2 substrates phospho-Rabs and Rab12 treated with MLi-2 was used to assess active LRRK2 in iPSC-DA neuronal cultures (36) (Supplementary Material, Fig. S5F and I). MLi-2 treatment (48 h, 100 nm) consistently decreased phosphorylation of phospho-Rabs and pRab12/Rab12 ratio in control iPSC-DA neuronal cultures, as expected (*P* = 0.0095 and 0.0457, respectively; Supplementary Material, Fig. S5G and I). Treatment with MLi-2 induced a decrease in phospho-Rabs immunoblot signal, and a non-significant decrease in phosphorylated pRab12 substrate, in *LRRK2^R1441C^* iPSC-DA neuronal cultures (*P* = 0.0088 and *P* = 0.0610, respectively; Supplementary Material, Fig. S5G and I).

**Figure 2:**
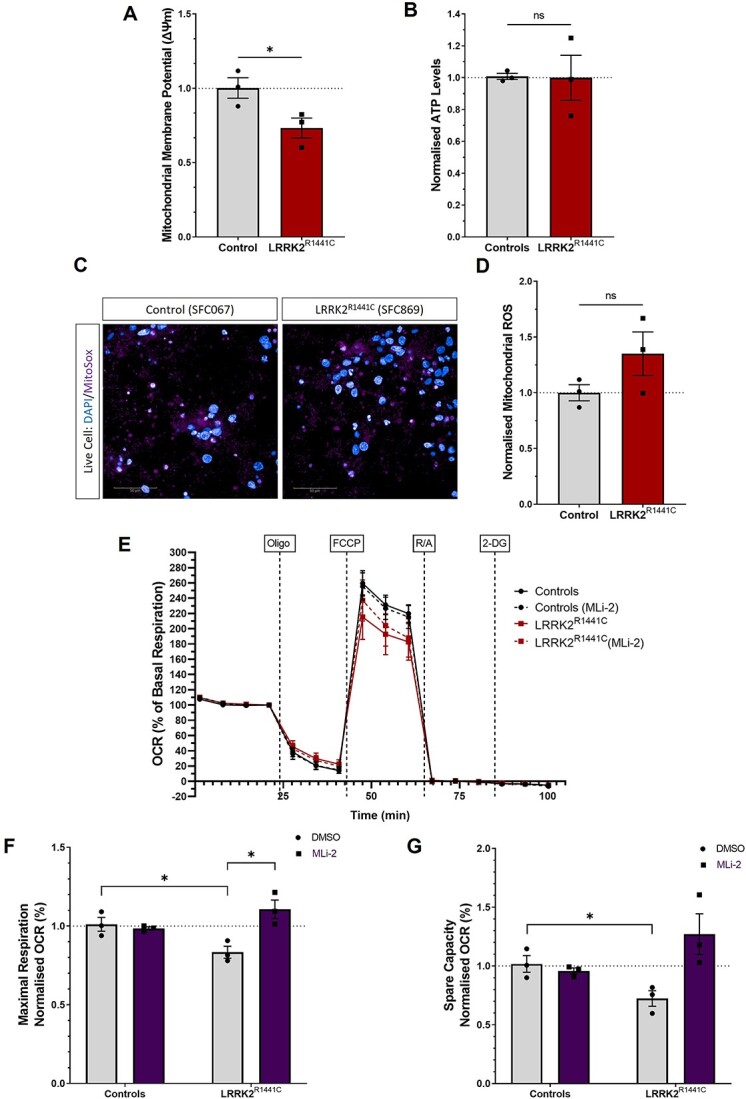
Mitochondrial dysfunction in Parkinson’s iPSC-DA neuronal cultures carrying the *LRRK2^R1441C^* mutation. iPSC-DA neuronal cultures from controls and *LRRK2^R1441C^* Parkinson’s patient lines were generated to assess (**A**) mitochondrial membrane potential changes measured with JC-10 and (**B**) ATP production measured using a luminescent assay and normalized to number of nuclei. Data points represent genotype lines, averaged by number of differentiations. Error bars: mean ± SEM from three independent differentiations (ns: non-significant; ^*^P < 0.05; unpaired *t* test). (**C**) Oxidative stress was measured as abundance of mitochondrial ROS superoxide anion (O_2_^·−^), as revealed by MitoSox fluorescent puncta number per cell (**D**). Error bars, mean ± SEM from four independent differentiations (ns: non-significant; unpaired *t* test). (**E**) Oxygen consumption rates (OCRs) in iPSC-DA neuronal cultures treated with DMSO or MLi-2 (100 nm) for 48 h were measured on an XF24 Analyzer. Normalized OCR profiles (% OCR to basal OCR) for control and *LRRK2^R1441C^* iPSC-DA neuronal cultures. (**F**) Maximal respiration was calculated as measurements after FCCP injection, normalized from OCR %, and fold change from DMSO-treated controls was quantified to assess MLi-2 effect. (**G**) Spare capacity was calculated as the difference between maximal and basal respiration, normalized from OCR %, and fold change from DMSO-treated controls was quantified to assess MLi-2 effect. Data points represent genotype lines, averaged by number of differentiations. Error bars: mean ± SEM from three independent differentiations (ns: non-significant; ^*^P < 0.05; two-way ANOVA).

To understand the effects of the *LRRK2^R1441C^* mutation on mitochondrial health in dopaminergic neurons, we performed a number of assays of mitochondrial function. Consistent with observations in primary cortical neuronal cultures, *LRRK2^R1441C^* iPSC-DA neuronal cultures demonstrated lower mitochondrial membrane potential (ΔΨm) under basal conditions (*P* = 0.0490) (Fig. 2A) with no change in basal ATP levels (Fig. 2B). However, although iPSC-DA neuronal cultures derived from two lines demonstrated increased mitochondrial ROS production, as assessed by MitoSox, this effect was not significant in *LRRK2^R1441C^* iPSC-DA neuronal cultures overall (Fig. 2C and D).

LRRK2 kinase inhibitors have been described to rescue mitochondrial alterations in PD models (22,27,28). We first confirmed that LRRK2 kinase activity was efficiently inhibited by MLi-2 in iPSC-DA neuronal cultures after 48 h of treatment, as demonstrated by ~50% reduction in the phosphorylation of LRRK2 substrate phospho-Rabs and pRab12 (Supplementary Material, Fig. S5F–I). However, treatment of *LRRK2^R1441C^* or healthy control iPSC-DA neuronal cultures with MLi-2 had no effect on mitochondrial membrane potential under basal conditions (Supplementary Material, Fig. S6A).

To understand the effect of LRRK2 kinase activity on cellular response to mitochondrial stress, cultures were treated with the LRRK2 kinase inhibitor MLi-2 for 48 h, prior to depolarization with CCCP. Both control and *LRRK2^R1441C^* iPSC-DA neuronal cultures treated with CCCP for 6 h showed a significant decrease in mitochondrial membrane potential, as expected. Furthermore, MLi-2 treatment was not able to rescue mitochondrial membrane potential in control and *LRRK2^R1441C^* iPSC-DA neuronal cultures after CCCP-induced depolarization (Supplementary Material, Fig. S6B). Similarly, LRRK2 kinase inhibition was unable to rescue CCCP-induced decreases in ATP levels (Supplementary Material, Fig. S6C and D) or increased mitochondrial ROS production (Supplementary Material, Fig. S6E and F), regardless of genotype. Thus, MLi-2 treatment was not able to rescue mitochondrial health in CCCP-induced depolarizing conditions.

Next, we investigated if the observed changes in mitochondrial membrane potential were associated with impaired cellular oxygen consumption and glycolysis in iPSC-DA neuronal cultures. Basal oxygen consumption rate (OCR) was not significantly changed in *LRRK2^R1441C^* iPSC-DA neuronal cultures (*P* = 0.3084) (Supplementary Material, Fig. S7A). To assess the relative changes in cellular oxygen consumption in each line, OCRs were calculated relative to the basal respiration rates (Fig. 2E, Supplementary Material, Fig. S7B and C). Maximal respiration was decreased by approximately 20%, with spare capacity reduced by 25% in *LRRK2^R1441C^* iPSC-DA neuronal cultures, when compared with control cells (*P* = 0.0333 and 0.0385, respectively) (Fig. 2F and G). MLi-2 treatment significantly increased maximal respiration (*P* = 0.0139) and showed a non-significant increase in spare capacity (*P* = 0.0619) in *LRRK2^R1441C^* iPSC-DA neuronal cultures, which suggests a partial rescue of mitochondrial function (Fig. 2F and G). Measuring extracellular acidification rate to evaluate glycolysis showed no differences between genotypes (Supplementary Material, Fig. S7D–E). Taken together, these results show subtle changes in the mitochondrial function in patient *LRRK2^R1441C^* iPSC-derived DA neurons, including decreased mitochondrial membrane potential, decreased maximal respiration and mitochondrial spare capacity.

### PINK1-dependent pS65Ub and basal mitophagy levels are decreased in *LRRK2^R1441C^* iPSC-DA neuronal cultures

**Figure 3:**
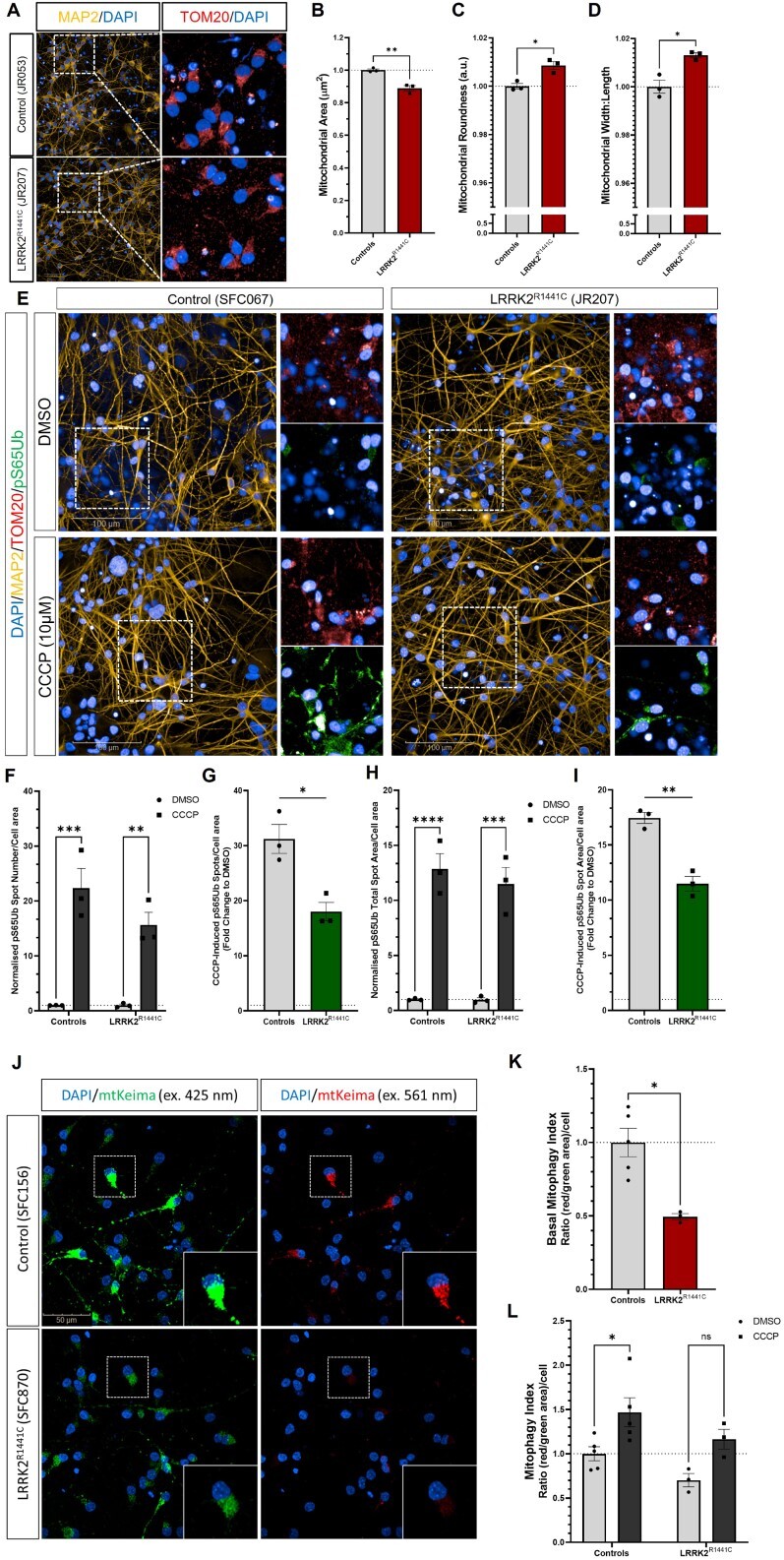
PINK1-dependent pS65Ub formation and mitophagy levels are impaired in patient iPSC-DA neuronal cultures carrying the *LRRK2^R1441C^* mutation. iPSC-DA neuronal cultures from control and *LRRK2^R1441C^* patient lines were fixed, stained for MAP2, pS65Ub and TOM20. (**A**) TOM20 puncta were identified per neuronal area (MAP2^+^) and measured to assess mitochondrial morphology in control and *LRRK2^R1441C^* iPSC-DA neuronal cultures. The measurements for mitochondrial area (μm^2^) (**B**), mitochondrial roundness (**C**) and mitochondrial width to length (**D**) were compared between genotypes. Data points represent genotype lines, averaged by number of differentiations. Error bars: mean ± SEM from five independent differentiations (ns: non-significant; ^*^P < 0.05; ^*^^*^P < 0.01; unpaired *t* test). (**E**) pS65Ub puncta were quantified as fold change from basal (DMSO) after treatment with 10 μm CCCP for 6 h and total spot number (**F**), and total spot area (**H**) were measured per neuronal area (MAP2^+^) and compared between genotypes (ns: non-significant; ^*^P < 0.05; ^*^^*^P < 0.01; ^*^^*^^*^P < 0.001; ^*^^*^^*^^*^P < 0.0001; two-way ANOVA). CCCP-induced pS65Ub total spot number (**G**) and total spot area (**I**) fold change to DMSO was measured and compared between genotypes. Data points represent genotype lines, averaged by number of differentiations. Bars, mean ± SEM from three independent differentiations (ns: non-significant; ^*^P < 0.05; unpaired *t* test). (**J**) iPSC-derived DA neuronal cultures were transduced with dox-inducible mt-mKeima lentivirus reporter and treated with doxycycline (1 μg/mL, 48 h). (**K**) The mitophagy index was quantified using the ratio of mt-mKeima total red area (ex. 561 nm)/mt-mKeima total green area (ex. 421 nm) per cell under basal conditions. Data points represent genotype lines, averaged by number of differentiations. Error bars, mean ± SEM from two to four independent differentiations (^*^P < 0.05; unpaired *t* test). (**L**) Quantification of mt-mKeima mitophagy index after treatment with CCCP (5 μm, 6 h). Data points represent genotype lines, averaged by number of differentiations. Error bars, mean ± SEM from three independent differentiations (^*^P < 0.05; two-way ANOVA).

To further investigate the effects of the *LRRK2^R144C^* mutation on mitochondria in iPSC-DA neurons and to understand the cell-type-specific effects of the mutation, we assessed mitochondrial morphology in patient and control iPSC-DA neuronal cultures by immunostaining for the protein TOM20 under basal conditions. We observed that mitochondria were significantly smaller, rounder and more fragmented in *LRRK2^R1441C^* iPSC-DA neuronal cultures (Fig. 3A–D). Furthermore, we confirmed CCCP-induced fragmentation and depletion of the mitochondrial network in both control and *LRRK2^R1441C^* iPSC-DA neuronal cultures (Supplementary Material, Fig. S8A–C).

Given these observations of changes in mitochondrial morphology in patient *LRRK2^R1441C^* iPSC-DA neuronal cultures, and our observations that *LRRK2^R1441C^* primary cortical cultures demonstrated decreased mitophagy initiation through impaired PINK1-dependent pS65Ub levels upon mitochondrial depolarization (Fig. 2), we assessed depolarization-induced pS65Ub in iPSC-DA neuronal cultures from *LRRK2^R1441C^* patients. Co-immunostaining of pS65Ub and TOM20 allowed us to assess the mitochondria labeled for mitophagy in *LRRK2^R1441C^* and control iPSC-DA neuronal cultures following mitochondrial depolarization (Fig. 3E). While CCCP induced a large increase in both the number of pS65Ub spots and pS65Ub spot area in iPSC-DA neuronal cultures (controls: *P* = 0.0002 and *P* < 0.0001; *LRRK2^R1441C^*: *P* = 0.0025 and *P* = 0.0002, for pS65Ub spot number and area, respectively) (Fig. 3F and H), the fold induction of pS65Ub after depolarization was decreased in *LRRK2^R1441C^* iPSC-DA neuronal cultures (*P* = 0.0132 and *P* = 0.0020 for spot number and area, respectively) (Fig. 3G and I). These deficits in pS65Ub induction in *LRRK2^R1441C^* neurons were not rescued by LRRK2 kinase inhibition by MLi-2 (Supplementary Material, Fig. S8D and E).

Finally, we measured mitophagy in iPSC-DA neuronal cultures using a doxycycline (dox)-inducible *mt-mKeima* live reporter (37), which was efficiently transduced into these cells (Fig. 3J). Consistent with our results for the *LRRK2^R1441C^* primary cortical cultures, patient *LRRK2^R1441C^* iPSC-DA neuronal cultures demonstrated a ~50% impairment in basal mitophagy (*P* = 0.0357) compared with healthy control iPSC-DA neuronal cultures (Fig. 3K). The non-acidic signal from the *mt-mKeima* reporter also allowed assessment of mitochondrial morphology, which, in agreement with the TOM20 immunostaining, demonstrated increased mitochondrial fragmentation in *LRRK2^R1441C^* neurons (Fig. 3A–D, Supplementary Material, Fig. S8F–H). Treatment with CCCP (5 μm, 6 h) induced mitophagy in both genotypes, although this reached significance only for the controls (*P* = 0.0257) (Fig. 3L, Supplementary Material, Fig. S8I). Consistent with our observations in primary cortical cultures, these data indicate that the activation of PINK1-dependent mitophagy marker pS65Ub is impaired and mitophagy levels are decreased in patient *LRRK2^R1441C^* neurons, independent of LRRK2 kinase activity.

### MIRO1 degradation is impaired in *LRRK2^R1441C^* iPSC-DA neuronal cultures

As accumulation of the PINK1-substrate pS65Ub, an early event in mitophagy, and basal mitophagy levels were impaired in *LRRK2^R1441C^* iPSC-DA neuronal cultures, we assessed the molecular mechanism behind this cellular phenotype. Several mechanisms have been proposed to link LRRK2 and regulation of mitophagy through the impaired degradation of MIRO1 and increased phosphorylation of Rab10 (27,30). No difference in Rab10 phosphorylation was observed following mitochondrial depolarization (Supplementary Material, Fig. S9). Given the observed impairment in early mitophagy initiation, we investigated the role of MIRO1 in depolarization-induced mitophagy. This mitophagy regulatory mechanism has been described to rely on LRRK2 and MIRO1 forming a complex after mitochondrial depolarization, which is independent of LRRK2 kinase-mediated phosphorylation (30).

**Figure 4:**
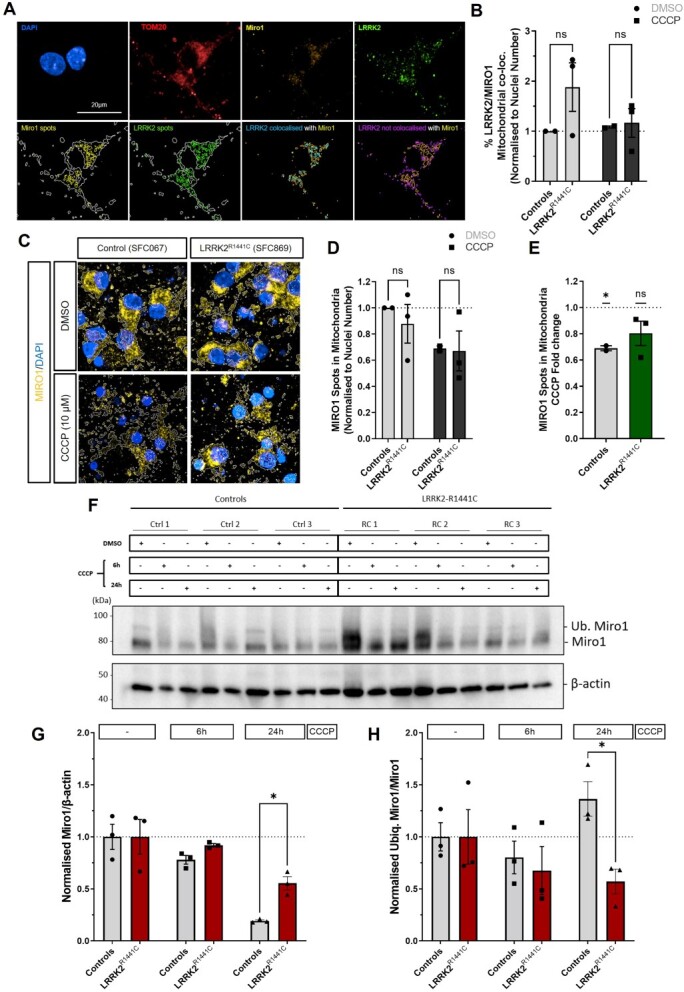
The *LRRK2^R1441C^* mutation impairs MIRO1 degradation. (**A**) To investigate interaction of LRRK2 and MIRO1 at mitochondria, LRRK2 and MIRO1 were co-immunostained under basal conditions in *LRRK2^R1441C^* and control iPSC-DA neuronal cultures. An example from control iPSC-DA neurons (SFC067) illustrates MIRO1 and LRRK2 co-localization within mitochondria (TOM20) and shows that the analysis pipeline identifies mitochondrial staining (TOM20) as the region of interest, then finds total MIRO1 spots within mitochondrial regions (yellow spots within white border) and total LRRK2 spots within mitochondrial regions (green spots within white border). Finally, the pipeline identifies all LRRK2 spots co-localized with MIRO1 within mitochondrial regions (cyan blue spots) and all the LRRK2 spots not co-localized with MIRO1 within mitochondrial regions (purple spots). (**B**) Quantification of the LRRK2 spots co-localized with MIRO1 within mitochondrial regions, as a percentage of all LRRK2 spots, normalized to the number of nuclei per well. (**C**) MIRO1 spots within mitochondrial regions, defined by TOM20 staining (yellow spots within white border) from *LRRK2^R1441C^* and control iPSC-DA neuronal cultures. (**D**) Quantification of MIRO1 spots within mitochondria, with DMSO and CCCP treatment, normalized to the number of nuclei per well, compared with DMSO control levels. (**E**) CCCP fold change compared between controls and *LRRK2^R1441C^* iPSC-DA neuronal cultures. Protein lysates of iPSC-DA neuronal cultures from *LRRK2^R1441C^* patient and control lines after 6- or 24-h treatment with 10 μm CCCP were quantified by western blot to assess changes in (**F**, **G**) MIRO1 and (**F**, **H**) ubiquitinated MIRO1 levels. Data points represent fold change in protein levels after CCCP treatment per line, averaged by number of differentiations. Error bars: mean ± SEM from two independent differentiations (ns: non-significant; ^*^P < 0.05; two-way ANOVA). Data points represent genotype lines, averaged by number of differentiations. Bars, mean ± SEM from two independent differentiations (ns: non-significant; ^*^P < 0.05; two-way ANOVA; unpaired *t* test).

Consistent with this observation, we observed that LRRK2 and MIRO1 co-localize within mitochondrial network (TOM20 positive) regions (Fig. 4A, Supplementary Material, Fig. S10A–E). However, the percentage of LRRK2-MIRO1 co-localized within mitochondrial regions was not significantly different in *LRRK2^R1441C^* iPSC-DA neuronal cultures under basal or CCCP-induced conditions (Fig. 4B), in contrast to previous findings that reported a decreased LRRK2^G2019S^/MIRO1 interaction (30).

As expected, we observed a CCCP-dependent decrease in the number of mitochondria-localized MIRO1 events (spot number) by immunocytochemistry in both control and *LRRK2^R1441C^* iPSC-DAn (Fig. 4C–E). Importantly, we observed no differences in MIRO1 spots not co-localized with mitochondrial marker TOM20 upon CCCP-induced depolarization (Supplementary Material, Fig. S10A, F and G). Moreover, LRRK2 spots in mitochondria show no differences between genotypes or CCCP-treated conditions (Supplementary Material, Fig. S10H and I).

To further understand the effect of *LRRK2^R1441C^* on MIRO1 degradation, we treated iPSC-DA neuronal cultures with CCCP for 6 h and 24 h to induce mitochondrial depolarization and assessed levels of MIRO1 and MIRO1 ubiquitination in iPSC-DA lysates. In agreement with our results from immunocytochemistry, control iPSC-DA neuronal cultures exhibited decreased levels of MIRO1 expression after 24 h of treatment (Fig. 4F and G). However, the decrease in MIRO1 expression was impaired in *LRRK2^R1441C^* iPSC-DA neuronal cultures (*P* = 0.0441), demonstrating that MIRO1 removal from depolarized mitochondria is impaired in mutant *LRRK2^R1441C^* cells (Fig. 4G). Moreover, as the ubiquitination of MIRO1 is necessary for its degradation (38), we also quantified ubiquitinated MIRO1. Consistent with the impaired degradation of MIRO1, a decreased proportion of MIRO1 was found to be ubiquitinated in *LRRK2^R1441C^* iPSC-DA neuronal cultures 24 h after CCCP treatment compared with control (*P* = 0.0311) (Fig. 4H).

Overall, we demonstrate that *LRRK2^R1441C^* iPSC-DA neuronal cultures exhibit altered mitochondrial morphology and mitochondrial dysfunction. These deficits may be driven, in part, by impairments in the clearance of damaged mitochondria owing to impaired PINK1-dependent pS65Ub accumulation and impaired degradation of MIRO1 found in a complex with LRRK2 after mitochondrial depolarization.

### Comparing *LRRK2^R1441C^* patient lines reveals differences in mitochondrial health and mitophagy in iPSC-DA neuronal cultures

**Figure 5:**
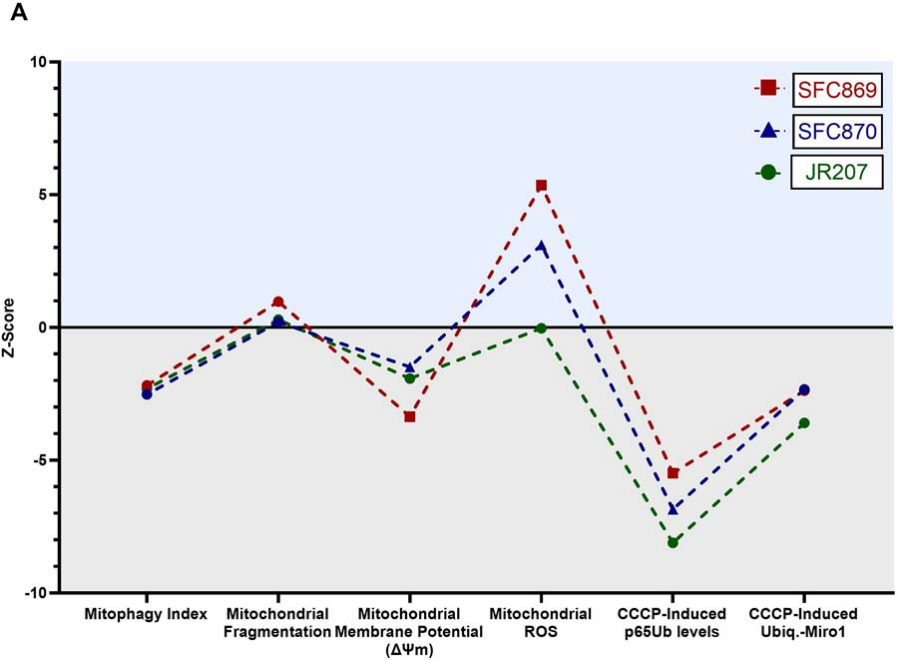
Heterogeneity in mitochondrial phenotypes among Parkinson’s *LRRK2^R1441C^* patient iPSC-DA neuronal cultures. (**A**) *Z*-scores were calculated for iPSC-DA neuronal cultures derived from each *LRRK2^R1441C^* Parkinson’s patient line plotted relative to control lines to assess inter-patient variation in mitophagy index levels, mitochondrial membrane potential (ΔΨm), mitochondrial reactive oxygen species (ROS), pS65Ub levels (fold change in total pS65Ub spot area after CCCP treatment) and ubiquitinated MIRO1 induction (fold change in Ubiq.-MIRO1 after CCCP treatment as assessed by immunoblot). Line SFC869 corresponds to patient line SFC869-03-04, SFC870 corresponds to patient line SFC870-03-05 and JR207 corresponds to patient line JR207-03.

iPSC-derived cellular models reflect the inherent variability between donors, making it essential to include lines from multiple patients in any study. To study the variability observed between iPSC-DA neuronal cultures derived from the three different *LRRK2^R1441C^* patient lines in some assays of mitochondrial health and mitophagy, we calculated the corresponding *Z*-scores per patient line for the altered mitochondrial phenotypes observed (Fig. 5). Reassuringly, we found consistent observations across phenotypes *within* each patient line in addition to the consistent phenotypes within LRRK2 genotypes. For example, all three *LRRK2^R1441C^* patient lines showed decreased basal mitophagy levels in iPSC-DA neuronal cultures, and the iPSC-DA neuronal cultures from *LRRK2^R1441C^* patient line SFC869 displayed the most fragmented mitochondria with the most decreased mitochondrial membrane potential, which corresponds to the highest mitochondrial ROS burden. Conversely, iPSC-DA neuronal cultures from *LRRK2^R1441C^* patient JR207 presented the least altered mitochondrial network and the most decreased levels of mitophagy activation among *LRRK2^R1441C^* patient lines.

## Discussion

Here, we have characterized mitochondrial health in two independent neuronal models of *LRRK2^R1441C^* and demonstrated mitochondrial dysfunction and impaired mitophagy activation and flux. First using transgenic primary cortical neuronal cultures, we observed that the *LRRK2^R1441C^* mutation decreases mitochondrial membrane potential and increases ROS production. This mitochondrial dysregulation is linked to a functional impairment of mitophagy, both under basal and depolarizing conditions. Interestingly, no significant changes were found in *LRRK2^G2019S^* cortical neuronal cultures, suggesting divergent pathology of *LRRK2* mutations (Fig. 1). Second, similar perturbations in mitochondrial function were demonstrated in human *LRRK2^R1441C^* iPSC-DA neuronal cultures, revealing decreased mitochondrial membrane potential, decreased maximal respiration and spare capacity in *LRRK2^R1441C^*. In parallel, we observed reduced pS65Ub levels in *LRRK2^R1441C^* iPSC-DA neuronal cultures. Interestingly, all *LRRK2^R1441C^* phenotypic alterations were shown to be independent of LRRK2 kinase activity. We have investigated previously reported mechanisms by which LRRK2 regulates mitophagy, including altered interaction of mutant LRRK2 with MIRO1, altered Drp1 and exacerbated phosphorylation of Rab10 (22,27,30). We demonstrate here that LRRK2 and MIRO1 proteins co-localize within mitochondria and that the *LRRK2^R1441C^* mutation results in no significant disruption of that interaction.

Mitochondrial dysfunction has been thoroughly described in *LRRK2^G2019S^* PD models to date, with the more penetrant but less frequent *LRRK2^R1441C^* mutation less explored. Here, we show that *LRRK2^R1441C^* iPSC-DA neuronal cultures display decreased mitochondrial membrane potential, reduced maximal respiration and spare capacity, indicating that mitochondria are less efficient when compared with control iPSC-DA neuronal cultures (Fig. 2). We observed specific changes in membrane potential and OXPHOS function in *LRRK2^R1441C^* iPSC-DA neuronal cultures although there was no gross energetic deficit. However, ATP levels in *LRRK2^R1441C^* iPSC-DA neuronal cultures were unchanged, suggesting compensation by other pathways. It has been previously reported that mitochondrial uncoupling proteins are altered in *LRRK2^G2019S^* models and similar mechanisms may also be operative in R1441C neurons (39).

Furthermore, calculating *LRRK2^R1441C^* patient line *Z*-scores for the different assays in our study revealed that DA neuronal cultures derived from the *LRRK2^R1441C^* iPSC line SFC869 presented with the worst *Z*-score for mitochondrial morphology, mitochondrial membrane potential and mitochondrial ROS, while presenting the highest score for mitophagy activation of all the patient lines. Overall, it is clear that the *LRRK2^R1441C^* patient line SFC869 displayed more severe phenotypical alterations than iPSC-DA neurons derived from patient lines SFC870 and JR207 (Fig. 5). Although the present study does not establish causality between mitochondria damage and mitophagy, our work demonstrates how mitochondrial networks are intricately balanced and highlights inter-patient variability between *LRRK2^R1441C^* mutation carriers. The link between iPSC-DA neuronal mitochondrial phenotypes and patient clinical phenotypes will be interesting to understand in large iPSC collections from well-characterized patient cohorts (40). However, the lack of correction of *LRRK2^R1441C^* phenotypes in patient iPSC-DA neuronal cultures reported here suggests mechanisms independent of kinase activity.

We also observe changes in mitochondrial morphology in *LRRK2^R1441C^* iPSC-DA neuronal cultures. Specifically, mitochondria are smaller, rounder and have an increased width-to-length ratio, which indicates that mitochondria are fragmented. Our results suggest that damaged mitochondria are accumulating in iPSC-DA neuronal cultures (Fig. 3). Moreover, we report that *LRRK2^R1441C^* iPSC-DA neuronal cultures exhibit altered OXPHOS complex activity, confirming that mitochondrial morphology alterations are associated with mitochondrial dysfunction. When analyzing mitochondrial morphology in primary cortical cultures and in aged striatal tissue, there were no differences in *LRRK2^R1441C^* compared with nTG littermates, suggesting that PD-related mitochondrial morphology alterations could be cell-type specific.

Taken together, our work illustrates that mitochondria are damaged and less efficient in *LRRK2^R1441C^* neuronal cultures and mitochondria clearance through mitophagy is impaired. Removal of damaged mitochondria by mitophagy is a key step in limiting the pool of damaged mitochondria and failure of this process is linked to PD (41). We have shown that *LRRK2^R1441C^* primary cortical neurons exhibit decreased mitophagy levels under steady state and CCCP-induced depolarizing conditions, in line with previous reports from *LRRK2^G2019S^* models (28,33). We also confirm decreased levels of pS65Ub, which is induced by PINK1 activation, after mitochondrial depolarization in *LRRK2^R1441C^* iPSC-DA neuronal cultures. Importantly, the mechanism of decreased pS65Ub levels in *LRRK2^R1441C^* mutants could involve altered PINK1 activation or increased activity of ubiquitin phosphatases such as PPEF2 or PTEN-L (42,43). Therefore, decreased mitophagy could further exacerbate perturbations on mitochondrial homeostasis because damaged mitochondria are not being degraded and could disturb the mitochondrial network, contributing to molecular pathology in PD.

Investigating the mechanisms of LRRK2-mediated regulation of mitophagy, we were able to partially confirm the findings described by Hsieh *et al*. (30), whereby the interaction of LRRK2 with MIRO1 promotes MIRO1 degradation and removal from the OMM, leading to mitophagy initiation. In the present study, we demonstrate that MIRO1 depletion induced by mitochondrial damage is impaired in *LRRK2^R1441C^* iPSC-DA neuronal cultures. Importantly, we show that LRRK2-MIRO1 co-localization occurs within mitochondrial structures in iPSC-DA neuronal cultures but, contrary to the mechanism demonstrated for LRRK2^G2019S^/MIRO1 interaction, we do not observe a significant disruption of LRRK2^R1441C^/MIRO1 co-localization compared with control (Fig. 4). This evidence adds to the ongoing hypothesis that the underlying pathophysiology of the LRRK2^G2019S^ and LRRK2^R1441C^ mutations have distinct mechanisms impacting neuronal cell health. These observations are interesting when considering the multi-factored susceptibility of dopaminergic neurons of the *SNpc*.

Conversely, in iPSC-DA *LRRK2^R1441C^* neuronal cultures, we did not observe the increased phosphorylation of Rab10 found in *LRRK2^G2019S^* mutant primary fibroblasts. The differing results highlight the complex cell-type and mutation specificity of LRRK2-PD pathology. In a recent study, Hsieh *et al*. (44) demonstrated that small molecule modulators promoting MIRO1 degradation increased mitophagy levels in *LRRK2^G2019S^* iPSC-DA neuronal cultures (44), which may also be relevant in *LRRK2^R1441C^* models.

Overall, this study determined that *LRRK2^R1441C^* iPSC-DA neuronal cultures exhibited mitochondrial dysfunction and impaired mitophagy. We found that the LRRK2 kinase inhibitor MLi-2 did not fully restore OXPHOS function, mitochondrial membrane potential or CCCP-induced mitophagy activation defects in *LRRK2^R1441C^* iPSC-DA neuronal cultures. Importantly, different *LRRK2^R1441C^* patient iPSC-DA neuronal cultures displayed different phenotypic severities at the molecular level, reflecting a need for patient stratification in the treatment of LRRK2-related PD.

## Materials and Methods

### Plasmids, pharmacological agents, treatments and antibodies

The pharmacological agents used were 5 or 10 μm CCCP (Merck, C2759) for 6 h or 24 h (also 1.25, 2.5, 5, 10, 20 and 40 μm for dose-response treatments), and 100 nm MLi-2 (Tocris, 5756) for 48 h.

The primary antibodies used for western blotting (WB) or for immunocytochemistry (ICC) were rabbit anti-LRRK2 (WB, 1:100; Abcam ab133474), rabbit anti-LRRK2 (phospho S1292; WB, 1:100; Abcam ab203181) mouse anti-MIRO1 (WB, 1:700; Merck, WH0055288M1), rabbit anti-RAB10 (WB, 1:1000; Cell Signaling Technology, 8127S), rabbit anti-RAB10 (phospho T73; WB, 1:250; Abcam, ab230261), rabbit anti-RAB12 (WB, 1:1000; Proteintech, 18 843-1-AP), rabbit anti-RAB12 (phospho S106; WB, 1:250; Abcam, ab256487), rabbit anti-RAB8A (phospho T72; WB, 1:100; Abcam, ab230260), rabbit anti-GFP (WB, 1:100; ThermoScientific A-11122), HRP-conjugated anti-β-actin (WB, 1:50 000, Biolegend, 643807), anti-phospho-ubiquitin Alexa Fluor-488 conjugate (phosphor Ser65; ICC, 1:500; Merck, ABS1513-I-AF488), mouse anti-TOM20 (ICC, 1:500; Santa Cruz Biotechnology, sc-17 764F-10), rat anti-TOMM20 (ICC, 1:250; Abcam, ab289670 EPR15581-39), chicken anti-MAP2 (ICC, 1:1000; Abcam, ab92434), rabbit anti-TH (ICC, 1:500; Millipore, AB152), rabbit anti-MIRO1 (ICC, 1:100; Merck, HPA010687) and rabbit anti-LRRK2 (ICC, 1:100; Antibodies Inc., 75-253S). Peroxidase-linked secondary antibodies used for WB were goat anti-mouse IgG (H + L)–HRP conjugate (1706516) and goat anti-rabbit IgG (H + L)–HRP conjugate (1706515) (BioRad). Secondary antibodies for ICC were donkey anti-mouse and anti-rabbit Alexa Fluor-488 and -555 (Thermo Fisher, a21202, a31570, a21206, a31572), donkey anti-rabbit Alexa Fluor 647 (Invitrogen, a31573), goat anti-chicken Alexa Fluor-555 (Invitrogen a32932), donkey anti-rat Alexa Fluor 594 (Invitrogen, a21209) and donkey anti-mouse Alexa Fluor-647 (Thermo Fisher, a31571).

The Mito-QC construct (32) was kindly provided by Dr Ian Ganley (School of Life Sciences, The University of Dundee, Dundee, Scotland).

The pLEX-TRE3G-mt-mKeima-pPGK-rtTA3 vector (I8) was generated by sub-cloning an MTS-mKeima cassette into a pLEX backbone featuring a bi-directional promoter region centered on a transcription blocker. The MTS-mKeima cassette was generated by PCR of mt-mKeima (37) and subsequently inserted into the donor plasmid via InFusion HD (Cat# 639649) with 15 bp homology overlaps after digestion with NotI and HpaI, aligning the cassette with the TRE3G promotor.

### Animal husbandry

Sprague–Dawley (SD) rats were maintained in accordance with UK Home Office regulations, under the Animals (Scientific Procedures) Act of 1986. Animals had *ad libitum* access to food and water. Previously described *LRRK2*-expressing BAC transgenic rats were housed with littermate controls (31). Animals were fed RM1 diet (Special Diets Services). Room atmosphere was maintained at 22°C and 60–70% humidity, and animals were kept in a 12 h light/dark cycle. Both sexes were used throughout this study.

### Cell culture and transduction

#### iPSC-derived dopaminergic neurons

The generation and characterization of the iPSC lines from healthy individuals and from PD patients carrying the *LRRK2^R1441C^* mutation has been previously described (15,45). The *LRRK2-KO* line was also previously generated and characterized (34). Feeder-free iPSC lines were routinely cultured on Matrigel (BD Biosciences) in mTeSR1 (StemCell Technologies). Differentiation of dopaminergic neurons was based on previously described protocols (46) with modifications adapted from Fedele and colleagues (47).

Briefly, 2 days prior to neuronal induction, iPSCs were dissociated to single cells and plated at a density of 1.5 × 10^5^ cells/cm^2^ on Geltrex (Life Technologies). iPSCs were patterned for 10 days to become ventral midbrain precursor cells (46). After patterning (DIV 10), cells were expanded for 19 days in KSR and NNB medium (1:3) supplemented with LDN193189 (100 nm; Merck) and CHIR99021 (3 μm, Tocris) (47). Cells were passaged four times 1:2 with Accutase on days 10 + 1, 10 + 7, 10 + 13 and 10 + 18 and plated with 10 μm Y-27632 onto Geltrex-coated plates. Cells were frozen at DIV 10 + 18 in 1:3 of KSR/NNB medium supplemented with 10% (v/v) DMSO.

Following the expansion of ventral midbrain precursor cells (DIV 11), media was changed to NB medium containing Neurobasal medium, B27 and 2 mm l-glutamine (Life Technologies) and differentiated into dopaminergic neuronal cultures (46). Cells were replated at DIV 20 and seeded at 2.5 × 10^5^ cells/cm^2^, followed by 15 days of maturation. All experiments were carried out at DIV 35 and data were gathered from two to three independent differentiations.

#### Generation of rat cortical primary cultures

Transgenic male rats were bred with a Sprague–Dawley female to produce transgenic and non-transgenic offspring within the same litters. P1 rats were tail clipped and marked for genotyping. A minimum of three animals per genotype were pooled for each experiment. Tail clips were lysed to extract DNA and genotyped. Genotyped pups were culled (P2–P5) in accordance with UK Home Office regulations, under the Animals (Scientific Procedures) Act of 1986. Brains were extracted and placed in Neurobasal(A) media for transportation. Meninges were removed and cortical areas were dissected and shredded into ~0.5 mm pieces in a sterile Petri dish with sterile scalpels and collected in ice-cold, fresh Neurobasal(A) media. Tissue was incubated in a water bath at 37°C in Neurobasal(A) medium (Life Technologies) plus 0.1% trypsin, for 20 min. Trypsin was inactivated with Neurobasal(A) containing 20% fetal bovine serum (FBS) and samples were then centrifuged at 1000×*g* at 37°C for 5 min. FBS-containing media was then replaced with Neurobasal(A) wash media and centrifuged at 1000×*g* at 37°C for 3 min. The wash step was repeated one more time. Wash media was removed and replaced with growth media [Neurobasal(A) Plus, 2 mm l-glutamine, 1× B27, 1 mL MycoZap (Lonza)]. Tissue was triturated and cells were stained using Solution 18 (Chemotec) containing 80 μg/mL Acridine Orange and 40 μg/mL DAPI and counted using automated cell counter NucleoCounter NC-250 (ChemoMetec, Denmark). Cells were seeded at 5 × 10^5^ and kept in the incubator at 37°C, 5% CO_2_, 95% relative humidity and 50% of medium was replaced every 2 days. All assays were completed at DIV 14–15.

#### Mito-QC transduction

Primary cortical cultures were transduced with Mito-QC reporter lentivirus at a MOI of 2 immediately before plating on DIV 0 and the media was changed 24 h later. Experiments were performed at DIV 14.

#### Mt-mKeima transduction

iPSC-derived dopaminergic neurons (30K cells/well, 96 W half-area plate) were transduced with doxycyclin-inducible mt-mKeima lentivirus (MOI of 2) on replating D20. Neurons were further matured to D35–D50.

### Immunocytochemistry

Cells were washed, fixed with 4% paraformaldehyde solution for 20 min and then washed twice. Fixed cells were incubated with permeation buffer (10% v/v FBS, in PBS) for 15 min, followed by 1 h at room temperature in blocking solution (0.1% v/v Triton-X, 10% v/v normal goat serum, in PBS) to block non-specific binding. Then, cells were incubated with primary antibodies diluted in blocking solution at 4°C overnight. Cells were washed and incubated with secondary antibodies and DAPI (NucBlue Fixed Cell ReadyProbes Reagent, R37606; Invitrogen) and were diluted in blocking solution for 2 h. Cells were then washed and imaged.

### Immunofluorescence and confocal microscopy

Immunofluorescent stainings were imaged using a high-throughput confocal microscope equipped with a chamber with controlled temperature and CO_2_ concentration, for live cell imaging (Opera Phenix High Content Screening System; Perkin Elmer). For quantitative analysis of immunocytochemistry staining, images were analyzed using Harmony High-Content Imaging and Analysis Software (v. 4.9, Opera Phenix; Perkin Elmer). Pipelines were created to characterize and quantify neuronal populations and determine spot number, spot area and relative spot intensity (corrected), average mean per well. To analyze mitochondrial morphology, we created a pipeline to identify nuclei and *Mito-QC* transduced neuronal cells, then applied the proprietary SER ridge filter to analyze mitochondrial spots (mCherry-positive puncta) and extracted the morphology measurements for area, roundness, width, length and width/length ratio, which are automatically calculated.

### Electron microscopy

Electron microscopy was performed as previously described (15,48). Briefly, rats were terminally anesthetized and perfused with 4% PFA and 0.1% glutaraldehyde. Free-floating coronal brain sections were cut to 60 μm using a vibratome and then incubated with tyrosine hydroxylase primary antibody (anti-TH, IHC 1:1000; Chemicon AB152). Dopaminergic terminals were revealed by silver-intensified immunogold conjugated secondary antibodies (Nanoprobes) and samples were dehydrated and embedded in Durcapan ACM resin (Fluka). Serial sections of dorsal striatum ~50 nm were cut and collected onto copper grids. Using ImageJ, a circumference was drawn around individual mitochondria and area and roundness measurements were recorded (50–100 mitochondria were analyzed per animal). The formula for circularity is 4π(area/perimeter^2^), with a value of 1.0 indicating a perfect circle (49).

### Immunoblotting

Cells were washed with PBS and lysed using radioimmunoprecipitation buffer [50 mm Tris, 150 mm NaCl, 0.1% sodium dodecyl sulfate, 0.5.% Na deoxycholate, 1% Igepal (pH 7.4)] containing protease (cOmplete EDTA-free, Roche) and phosphatase (PhosSTOP, Roche) inhibitors. Cells were detached using a cell scraper and collected. Lysates were centrifuged at 10 000×*g* at 4°C for 10 min. Proteins were quantified using a bicinchonic acid assay and absorbance readings at 562 nm. Whole-cell lysates were denatured at 95°C for 5 min, separated by electrophoresis in a 4–15% Criterion Tris–HCl polyacrylamide gels (BioRad), and the proteins were then transferred to a polyvinylidene difluoride (PVDF) membrane (Trans-Blot Turbo Midi PVDF transfer packs; BioRad) using a Trans-Blot Turbo Transfer System (BioRad), in accordance to manufacturer’s instructions. Membranes were incubated in blocking solution [5% non-fat milk (Merck) in PBS/0.1% Tween-20 (PBS-T)] for 1 h at room temperature and incubated with primary antibody overnight at 4°C. Membranes were washed with and incubated in horseradish peroxidase (HRP)-conjugated secondary antibody (1:1000) (BioRad) in blocking solution for 1 h. Membranes were washed and developed using ECL solution (Millipore). Membranes were imaged using the Chemidoc Touch Imaging System (BioRad) and quantified using ImageLab (BioRad).

### Assessing mitochondrial health and function

#### Cellular oxygen consumption

iPSC-derived DA neuronal cultures were plated in a XF96 Polystyrene Cell Culture Microplate (Seahorse Bioscience) on day 20 and further matured until day 35 before analysis. On the day of the assay, the assay medium was prepared fresh using the XF Base Medium (Agilent Technologies) supplemented with 10 mm glucose (Merck), 1 mm sodium pyruvate (Merck) and 2 mm l-glutamine (Thermo Fisher Scientific). At least 1 h before the assay, the cells were washed once with the assay medium and then incubated at 37°C in a non-CO_2_ incubator. Three baseline recordings were made, followed by sequential injection of the ATP synthase inhibitor oligomycin (Oligo; Merck, 1 μm), the mitochondrial uncoupler *p*-triflouromethoxyphenylhydrazone (FCCP; Merck, 1 μm), the Complex I and III inhibitors Rotenone and Antimycin A (R/A; Merck, 5 μm) and the glucose analog 2-deoxyglucose (2-DG; Merck, 50 mm). Mitochondrial respiration and glycolytic activity of DA cultures were measured using a Seahorse XFe96 Analyzer (Agilent Technologies) and normalized to total well protein content.

#### ATP production measurement

Media was removed from plates and cells were mixed with CellTiter-Glo 3D Cell Viability Assay (Promega). Then, 100 μL of substrate medium was transferred to a 96-white plate and incubated for 25 min at room temperature, shielded from light. To measure ATP production, luminescence was measured using the multi-mode plate reader PHERAstar FSX (BMG Labtech) and measurements were normalized to cell number. Cell number was measured prior to the start of the assay by adding Hoechst nuclear marker (NucBlue Live ReadyProbes Reagent, R37605; Invitrogen) to cells and imaging live (Opera Phenix High Content Screening System; Perkin Elmer).

#### Mitochondrial reactive oxygen species

To detect mitochondrial reactive oxygen species (ROS), cells were incubated with 2.5 μm MitoSOX Red Mitochondrial Superoxide Indicator (Life Technologies, M36008) and nuclear marker Hoechst (NucBlue Live ReadyProbes Reagent, R37605; Invitrogen) for 10 min at 37°C with 5% CO_2_. Following a wash with growth media, cells were immediately imaged and MitoSox fluorescence was measured at excitation/emission maxima: 488/650–760 nm, using a confocal microscope (Opera Phenix High Content Screening System; Perkin Elmer).

#### Mitochondrial membrane potential

To detect changes in mitochondrial membrane potential (ΔΨm), cells were incubated with 5 μm JC-10 (AAT Bioquest, Inc.) at 37°C for 1 h. The fluorescence intensities were measured using the multi-mode plate reader PHERAstar FSX (BMG Labtech) by fluorescence excitation/emission maxima: 514/529 nm, monomer form, and 585/590 nm, aggregate form. To establish that the JC-10 signal was indicative of ΔΨm, experiments were terminated inducing a maximal mitochondrial depolarization by addition of 10 μm carbonyl cyanide 3-chlorophenylhydrazone (CCCP; Merck).

### Assessing mitophagy flux

#### Mito-QC assay in primary cortical cultures

Imaging and analysis of the Mito-QC reporter to assess mitophagy was performed as previously described (50). In brief, cells were imaged on an Opera Phenix High Content Screening System (Perkin Elmer) using the following channels: DAPI, eGFP and mCherry. Images were then processed and analyzed using Harmony software (Perkin Elmer). In brief, the Harmony analysis pipeline consisted of the following building blocks: Find Nuclei, Find Cytoplasm, Image calculation: GFP/mCherry ratio, Find Spots (Red only), Calculate spot properties/cell.

#### Mt-Keima assay in iPSC-DA neuronal cultures

iPSC-derived dopaminergic neurons were transduced with doxycyclin-inducible mt-mKeima lentivirus and treated with doxycycline for 48 h, 1 μg/mL final concentration from a 5000× DMSO stock and nuclear marker Hoechst (NucBlue Live ReadyProbes Reagent, R37605; Invitrogen). Images were taken with the Opera Phenix High Content Screening System (Perkin Elmer), using a 40× objective with mKeima excitation at 425 nm (green) and 561 nm (red) and emission at 570–630 nm, and the DAPI channel for identification of nuclei.

Images were analyzed using Harmony software (Perkin Elmer) to provide ratio images of red and green mt-mKeima signal and mitochondrial morphology (green mitochondria only). The Mitophagy Index is defined as the ratio of red/green total mt-mKeima area (37).

### Statistical analysis

Graphs and statistical significance for all data was generated using Prism GraphPad (v. 9.3.1.471) software. The results are expressed as mean ± SEM. Statistical tests used were one- or two-way analysis of variance test to evaluate the differences between groups. One-sample *t* test or multiple *t* tests were used to compare between genotypes. Values of *P* <0.05 were considered statistically significant.

## Supplementary Material

HMG-2022-CE-00398_R3-Williamson_Madureira_et_al_2022-Supplementary_Figure_Legends_ddad102

HMG-2022-CE-00398_R3-Williamson_Madureira_et_al_2022-Supplementary_Figures_ddad102
