# Supplementary material for: Mitochondrial dysfunction and mitophagy defects in LRRK2-R1441C Parkinson’s disease models
Source: Hum Mol Genet. 2023 Jun 29;32(18):2808–21. doi: 10.1093/hmg/ddad102 (PMC10481106; doi:10.1093/hmg/ddad102)
Supplement: HMG-2022-CE-00398_R3-Williamson_Madureira_et_al_2022-Supplementary_Figure_Legends_ddad102 [file hmg-2022-ce-00398_r3-williamson_madureira_et_al_2022-supplementary_figure_legends_ddad102.docx]

**Supplementary Fig. 1 – Mitochondrial health and mitophagy levels in primary cortical neuronal cultures from *LRRK2* transgenic rats.**

Primary cortical neuronal cultures were generated from P1-P5 pups and assayed at DIV 14. **(A)** Mitochondrial morphology was assessed using Mito-QC transduced cortical cultures, to calculate mitochondrial area (μm^2^) **(B)** and mitochondrial width to length **(C)**, normalised to non-transgenic littermates. **(D)** The full dataset (from Fig. 1C) for mitochondrial ROS, measured using MitoSox live dye and compared between the genotypes. Mitochondrial health assessed in *LRRK2^G2019S^* and *LRRK2^hWT^* cortical cultures using mitochondrial membrane potential changes measured with JC-10 live dye **(E, I)**, by ATP levels measured using a luminescent assay **(F, J**), and by mitochondrial ROS **(G, K)** normalised to non-transgenic littermate levels. Data points represent averaged transgenic genotypes. Error bars: mean ± SEM from 3-4 independent litters (ns: non-significant; ∗P <0.05; Mann-Whitney Unpaired t test). **(H, L)** Mitophagic flux was calculated as *mCherry*-only puncta for *Mito-QC* reporter transduced primary cortical cultures. Values were normalized to non-transgenic littermates and quantified to assess *mCherry* under basal conditions for *LRRK2^G2019S^* and *LRRK2^hWT^* genotypes. Error bars: mean ± SEM from three independent litters (ns: non-significant; ∗P <0.05; Mann-Whitney Unpaired t test).

**Supplementary Fig. 2 – Validation of a pS65Ub induction assay.**

Wild-type or *PINK1* KO SH-SY5Y cells were treated with 10 μM CCCP for 6h. **(A)** Representative images of cells stained for pS65Ub (green), actin (phalloidin; red) and nuclei (DAPI; blue). **(B)** Quantification of pS65Ub potentiation after CCCP treatment, bars represent mean pS65Ub levels, normalised to DMSO treated WT cells (N=3).

**Supplementary Fig. 3 – Mitochondrial morphology in striatal tissue from aged *LRRK2* rats.**

**(A)** Representative immunogold labelled dopaminergic terminals, scale bar represents 500 nm. Mitochondrial morphology of the dorsal striatum of 21-22 months old *LRRK2* BAC transgenic rats was assessed by calculating **(B)** mitochondrial area (μm^2^) and **(C)** mitochondrial roundness using ImageJ software. Data points represent values averaged from each animal. Scale bars correspond to 500 nm. Bars, mean ± SEM from three independent litters (ns: non-significant; ∗P <0.05; Kruskal–Wallis non-parametric ANOVA).

Data points represent averaged measures per animal analysed for each genotype. Bars, mean ± SEM from three independent litters (ns: non-significant; ∗P <0.05; Kruskal–Wallis non-parametric ANOVA).

**Supplementary Fig. 4 – Characterisation of iPSC-DA neuronal cultures.**

**(A)** Representative images of control and *LRRK2^R1441C^* iPSC-DA neuronal cultures used throughout this study stained for MAP2, TH and DAPI. Scale bar represents 50 µm. Quantification of the percentage of cells positive for MAP2^+^ within the total nuclei count **(B)** and percentage of cells positive for TH^+^ within the MAP2^+^ population **(C)**, compared between control and *LRRK2^R1441C^* iPSC-DA neuronal cultures. Data points represent genotype lines, averaged by number of differentiations. Error bars: mean ± SEM from three independent differentiations (ns: non-significant; ∗P <0.05; Unpaired t test). **(D)** Table describing patient and control lines used in the study.

**Supplementary Fig. 5 – LRRK2 kinase activity and MLi-2 treatment in iPSC-DA neuronal cultures.**

To assess LRRK2 expression and kinase inhibition, iPSC-DA neuronal cultures from healthy controls, *LRRK2-KO* and *LRRK2^R1441C^* lines were treated with DMSO or MLi-2 (100 nM) for 48 h. Western blots were immunoblotted for phosphorylated LRRK2 (S1292) (pLRRK2) **(A)**, total LRRK2 **(A, D)**, phospho-Rabs **(A, F)**, phosphorylated Rab12 and total Rab12 **(H)** and β-actin **(A, D, F, H)**. **(A)** shows a *LRRK-KO* validation panel**,** where pLRRK2/LRRK2 **(B)** and phospho-Rabs/β-actin ratios **(C)** were quantified and compared between treatment groups. LRRK2 expression was normalised to β-actin, normalised to average of controls and compared between genotypes **(D, E)**. Expression of phosphorylated Rabs was quantified as phospho-Rabs/β-actin **(F, G)** and phosphorylated Rab12/Rab12 ratios **(H, I)**, normalised to average of controls and compared between treatment groups. Data points represent genotype lines, averaged by number of differentiations. Error bars: mean ± SEM from two independent differentiations (ns: non-significant; ∗P <0.05; ∗∗P <0.01; Two-Way ANOVA, Unpaired t test).

**Supplementary Fig. 6 – Live cell mitochondrial assays in patient iPSC-DA neuronal cultures carrying the *LRRK2^R1441C^* mutation.**

To assess the effects of LRRK2 kinase inhibition on mitochondrial membrane potential, iPSC-DA neuronal cultures from control and *LRRK2^R1441C^* patient lines were treated with MLi-2 (100 nM) for 48 h prior to JC-10 assay **(A)** or treated with 10 µM CCCP for 6 hours and treated with MLi-2 (100 nM) for 48 h prior to JC-10 assay **(B)**. The fold change from the DMSO treatment of each genotype was quantified. Data points represent genotype lines, averaged by number of differentiations. Error bars: mean ± SEM from two and four independent differentiations, respectively (ns: non-significant; ∗P <0.05; ∗∗P <0.01; ∗∗∗P <0.001; ∗∗∗∗P <0.0001; Multiple t test, Bonferroni post hoc, two-way ANOVA). ATP production was measured using a luminescence assay and compared between control and *LRRK2^R1441C^* patient iPSC-DA neuronal cultures, as fold change from basal (DMSO) after treatment with 10 µM CCCP for 6 hours **(C)** or with 10 µM CCCP for 6 hours MLi-2 (100 nM) for 48 h prior to assay **(D)**. Data points represent genotype lines, averaged by number of differentiations. Error bars: mean ± SEM from three independent differentiations. (ns: non-significant; ∗P <0.05; Multiple t test, Bonferroni post hoc, two-way ANOVA). Oxidative stress was measured as abundance of mitochondrial ROS superoxide anion (O_2_^·−^), as revealed by MitoSox fluorescent puncta fold change after treatment with 10 µM CCCP for 6 hours **(E)** or with 10 µM CCCP for 6 hours MLi-2 (100 nM) for 48 h prior to assay **(F)** prior to assay**.** Data points represent genotype lines, averaged by number of differentiations. Error bars: mean ± SEM from four and three independent differentiations, respectively. (ns: non-significant; ∗P <0.05; ∗∗P <0.01; Multiple t test, Bonferroni post hoc, two-way ANOVA).

**Supplementary Fig. 7 – Measuring oxygen consumption and extracellular acidification rates in iPSC-DA neuronal cultures.**

Oxygen consumption rates (OCRs) in iPSC-DA neuronal cultures were measured on an XF24 Analyzer. Oxygen consumption profiles (pmol O_2_/min/µg of protein) are represented for controls and *LRRK2^R1441C^* iPSC-DA neuronal cultures. **(A)** Basal respiration was calculated as measurements before oligomycin injection, for controls and *LRRK2^R1441C^* iPSC-DA neuronal cultures (pmol O_2_/min/µg protein). After normalisation to % of Basal OCR, Proton leak, ATP linked to oxygen consumption, maximal respiration and spare capacity were calculated. **(B)** Proton leak was calculated as the measurements after oligomycin injection **(C)** ATP production linked to oxygen consumption was calculated as the measurements after oligomycin injection and measurement of basal respiration. Data points represent genotype lines, averaged by number of differentiations. Bars, mean ± SEM from three independent differentiations (ns: non-significant; ∗P <0.05; Unpaired t test). **(D)** Extracellular acidification rates (ECARs) in iPSC-DA cultures were measured on an XF24 Analyzer. Normalised Extracellular acidification profiles (% ECAR to Basal ECAR) are represented for control and *LRRK2^R1441C^* iPSC-DA neuronal cultures. **(E)** Normalised ECAR was calculated for control and *LRRK2^R1441C^* iPSC-DA cultures. Control and *LRRK2^R1441C^* iPSC-DA neuronal cultures were treated with MLi-2 (100 nM) for 48 h and normalised ECAR were calculated. Data points represent genotype lines, averaged by number of differentiations. Error bars: mean ± SEM from two independent differentiations (ns: non-significant; ∗P <0.05; Unpaired t test).

**Supplementary Fig. 8 – PINK1/Parkin activation and mitophagy assay validation in iPSC-DA neuronal cultures.**

To confirm CCCP-induced depolarization effect, cells were fixed, stained for MAP2, pS65Ub and TOM20, and confocal microscopy images acquired. Mitochondrial morphology was assessed by calculating **(A)** mitochondrial area (μm^2^), **(B)** roundness and **(C)** mitochondrial width to length, normalised to DMSO treated controls and compared between DMSO and CCCP treatment. To assess the effects of LRRK2 kinase inhibition, control and *LRRK2^R1441C^* iPSC-DA neuronal cultures were treated with 10 µM CCCP for 6 hours and treated with MLi-2 (100 nM) for 48 h. Normalised pS65Ub puncta number (to average of controls) was quantified, and total spot number **(D)** and total spot area **(E)** was measured per neuronal area (MAP2^+^) and compared between genotypes. Data points represent genotype lines, averaged by number of differentiations. Error bars, mean ± SEM from two independent differentiations (ns: non-significant; ∗P <0.05; ∗∗P <0.01; ∗∗∗P <0.001; ∗∗∗∗P <0.0001; two-way ANOVA). **(F)** iPSC-derived dopaminergic cultures were transduced with mt-mKeima lentivirus reporter and treated with 5 µM CCCP for 6 h. The mitophagy index was quantified using the ratio of mt-mKeima total red area (ex. 561 nm)/ mt-mKeima total green area (ex. 421 nm) per cell. Data points represent genotype lines, averaged by number of differentiations. Bars, mean ± SEM from three independent differentiations (∗P <0.05; Unpaired t test). Mt-mKeima green puncta representing healthy mitochondria were analysed to assess mitochondrial morphology in iPSC-derived dopaminergic cultures from controls and LRRK2^R1441C^. The measurements for mitochondrial area **(G)**, mitochondrial roundness **(H)** and mitochondrial width to length **(I)** were compared between genotypes under basal conditions. Data points represent genotype lines, averaged by number of differentiations. Bars, mean ± SEM from two to four independent differentiations (ns: non-significant; ∗P <0.05; ∗∗P <0.01; Unpaired t test).

**Supplementary Fig. 9 – Investigating Phosphorylated Rab10 LRRK2-regulated mitophagy mechanism using iPSC-DA neuronal cultures.**

Protein lysates were prepared from iPSC-DA neuronal cultures from control and *LRRK2^R1441C^* patient lines. Western blots for phosphorylated Rab10 (phospho ST73) and Rab10 expression were quantified to assess pRab10/Rab10 fold change from basal (DMSO) in response to treatment with 10 μM CCCP for 6 h or 24 h **(A, B).** Data points represent genotype lines, averaged by number of differentiations. Bars, mean ± SEM from two independent differentiations (ns: non-significant; ∗P <0.05; ∗∗P <0.01; two-way ANOVA).

**Supplementary Fig. 10 – iPSC-DA neuronal culture expression of LRRK2/MIRO1 and TOM20.**

**(A)** iPSC-DA neuronal cultures from control and LRRK2^R1441C^ patient lines were treated with DMSO or CCCP (24 h, 10 μM) then fixed, stained for LRRK2, TOM20 and MIRO1. **(B)** Example Z-stack profile of MIRO1-LRRK2-TOM20 co-localisation in control lines showing individual planes (LRRK2, green; MIRO1, red; co-localisation, cyan). **(C-E)** Correlation plot showing the brightest z-plane in co-immunofluorescence images for **(C)** MIRO1-TOM20, **(D)** LRRK2-TOM20 and **(E)** MIRO1-LRRK2. The plots demonstrate that the highest fluorescence intensity for each marker pair generally occur in the same plane. **(F)** Quantification of MIRO1 spots localised outside of mitochondrial regions, defined by TOM20 staining from *LRRK2^R1441C^* and control iPSC-DA neuronal cultures, treated with DMSO and CCCP, normalised to each well nuclei number, compared to DMSO Control levels and **(G)** CCCP-fold change compared between controls and *LRRK2^R1441C^* iPSC-DA neuronal cultures. **(H)** Quantification of LRRK2 spots within Mitochondria, with DMSO and CCCP treatment, normalised to each well nuclei number, compared to DMSO Control levels and **(I)** CCCP-fold change compared between controls and *LRRK2^R1441C^* iPSC-DA neuronal cultures. **(J)** Nuclei number (DAPI), averaged per differentiation from *LRRK2^R1441C^* and control iPSC-DA neuronal cultures, treated with DMSO and CCCP. Data points represent genotype lines, averaged by number of differentiations. Bars, mean ± SEM from two independent differentiations (ns: non-significant; two-way ANOVA; Unpaired t test).
