# Supplementary material for: Mitochondrial dysfunction and mitophagy defects in LRRK2-R1441C Parkinson’s disease models
Source: Hum Mol Genet. 2023 Jun 29;32(18):2808–21. doi: 10.1093/hmg/ddad102 (PMC10481106; doi:10.1093/hmg/ddad102)
Supplement: HMG-2022-CE-00398_R3-Williamson_Madureira_et_al_2022-Supplementary_Figures_ddad102 [file hmg-2022-ce-00398_r3-williamson_madureira_et_al_2022-supplementary_figures_ddad102.zip › HMG-2022-CE-00398_R3_-_Williamson,_Madureira_et_al_2022_-_Supplementary_Figures_ddad102.pptx]

## Slide 1
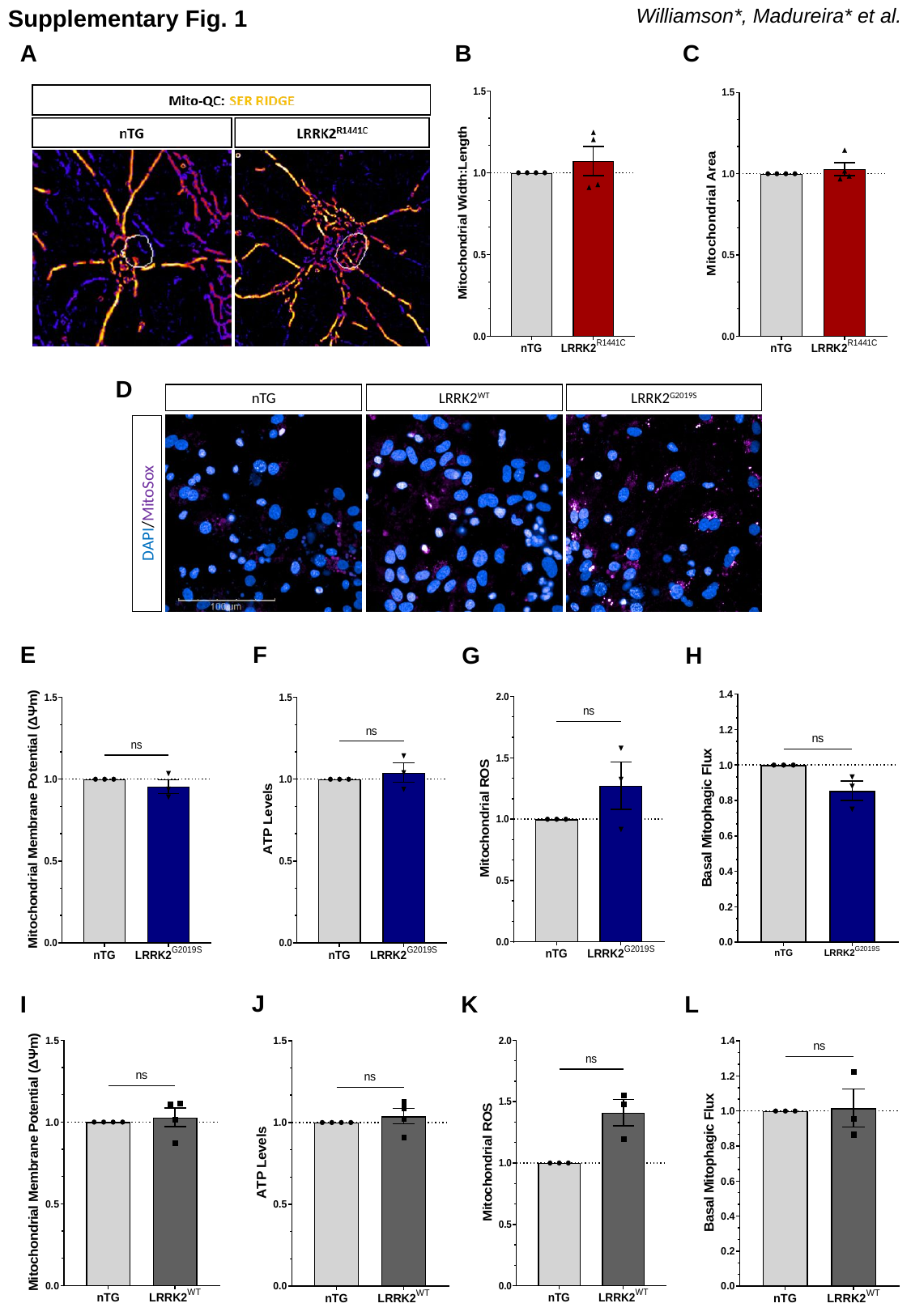

Williamson*, Madureira* et al.
Supplementary Fig. 1
C
A
B
D
nTG
LRRK2WT
LRRK2G2019S
DAPI/MitoSox
F
E
H
G
J
L
K
I

## Slide 2
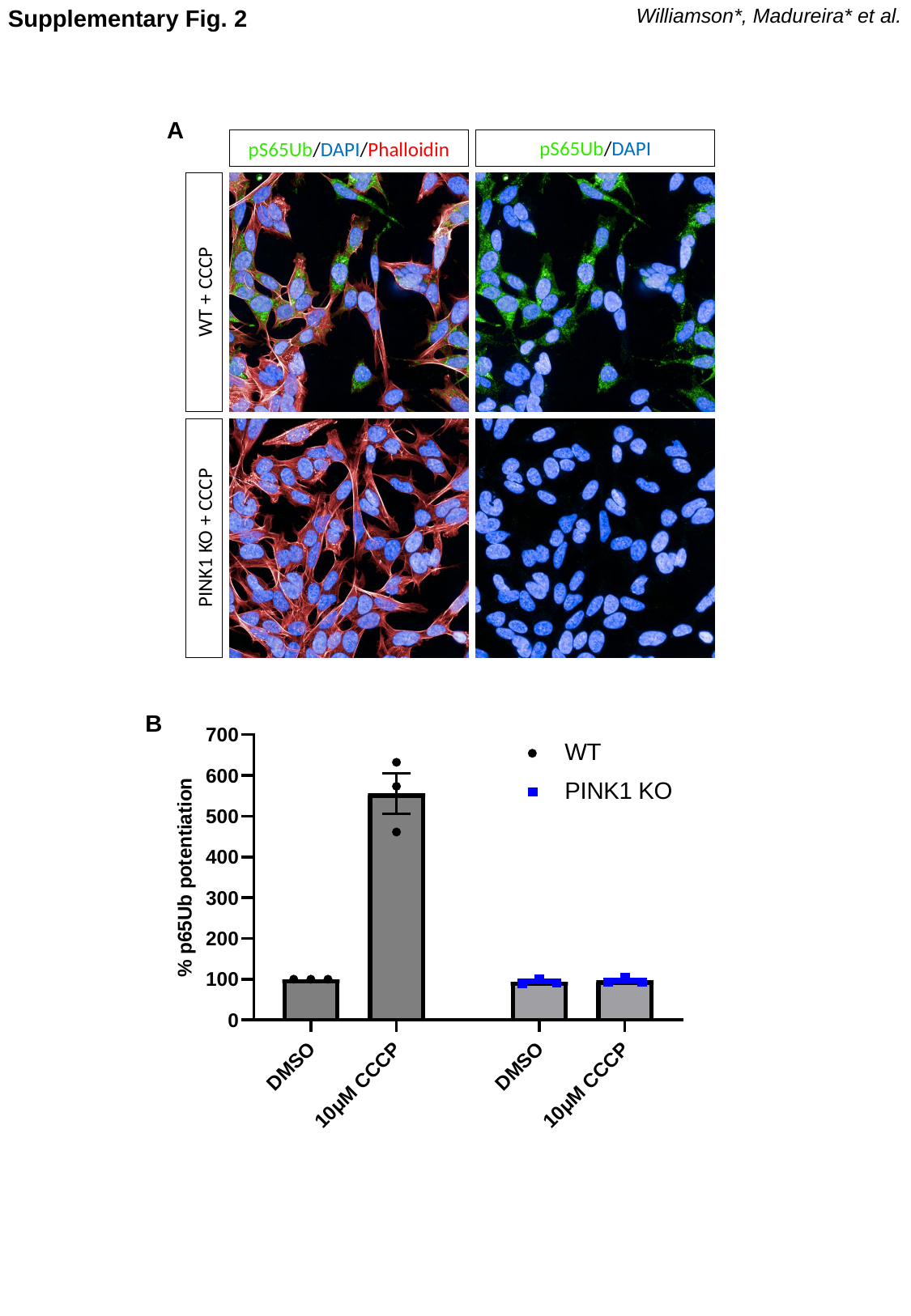

Williamson*, Madureira* et al.
Supplementary Fig. 2
A
pS65Ub/DAPI
pS65Ub/DAPI/Phalloidin
WT + CCCP
PINK1 KO + CCCP
B

## Slide 3
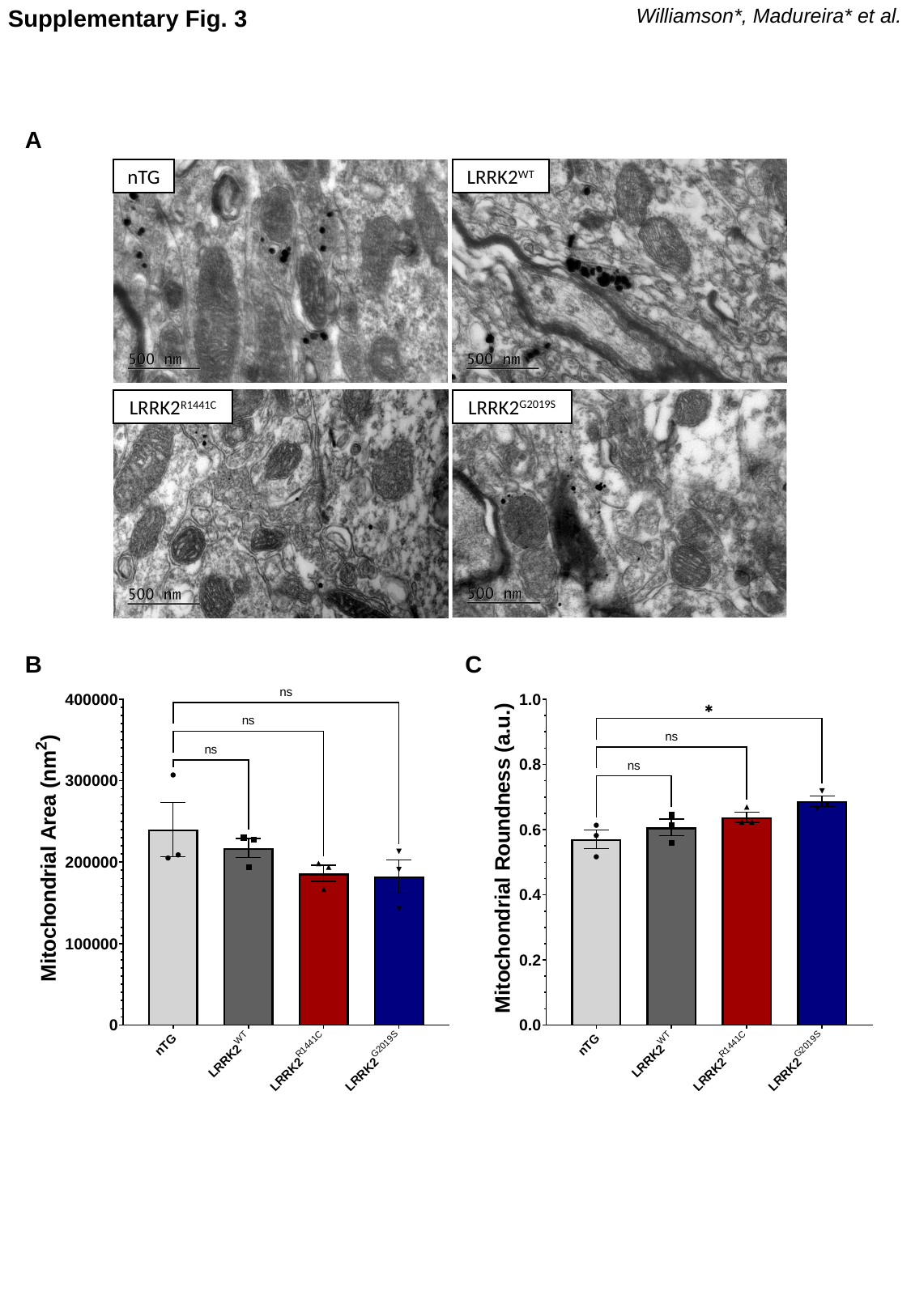

Williamson*, Madureira* et al.
Supplementary Fig. 3
A
LRRK2WT
nTG
LRRK2G2019S
LRRK2R1441C
B
C

## Slide 4
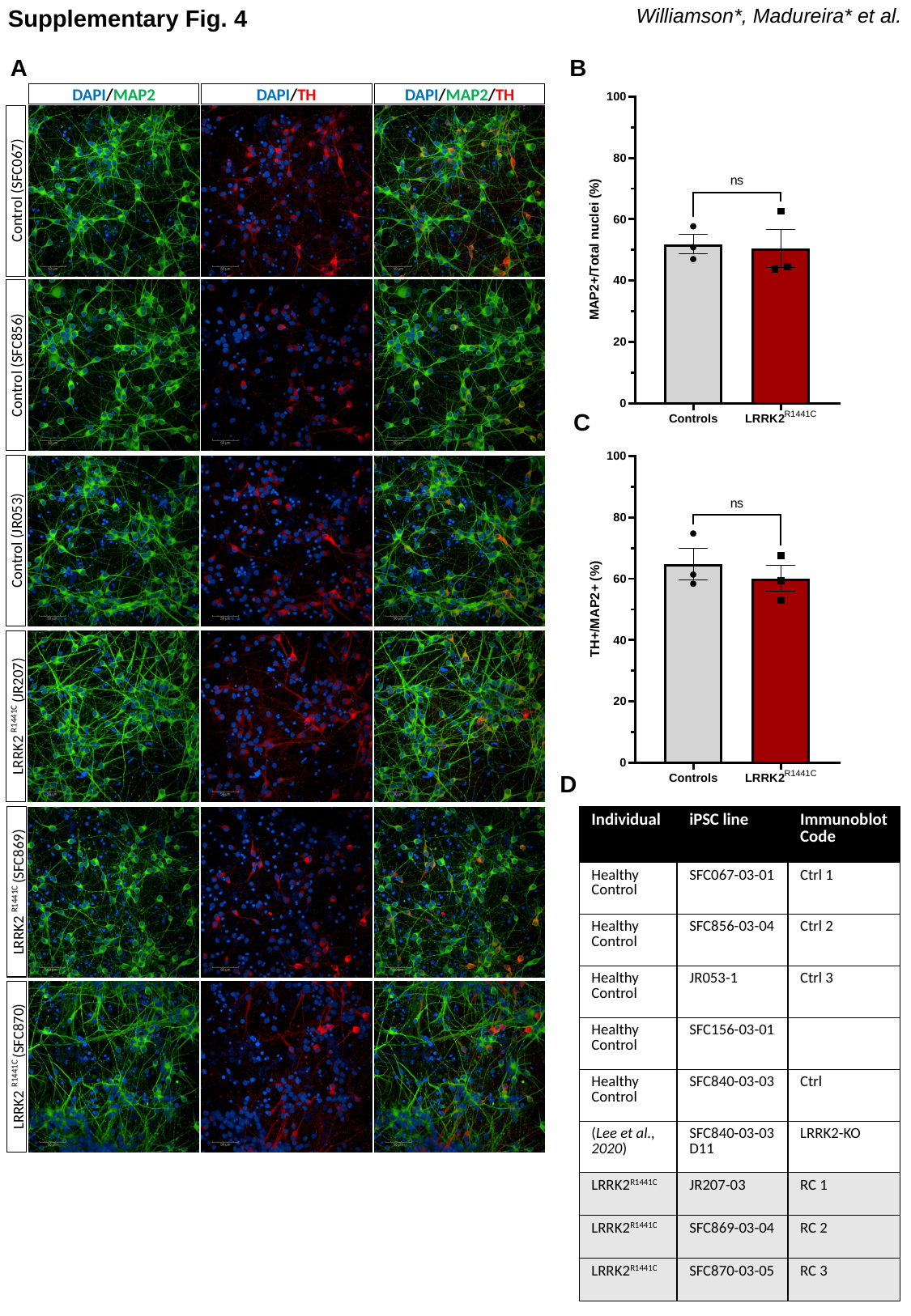

Williamson*, Madureira* et al.
Supplementary Fig. 4
A
B
DAPI/MAP2
DAPI/TH
DAPI/MAP2/TH
Control (SFC067)
Control (SFC856)
C
Control (JR053)
LRRK2 R1441C (JR207)
D
| Individual | iPSC line | Immunoblot Code |
| --- | --- | --- |
| Healthy Control | SFC067-03-01 | Ctrl 1 |
| Healthy Control | SFC856-03-04 | Ctrl 2 |
| Healthy Control | JR053-1 | Ctrl 3 |
| Healthy Control | SFC156-03-01 | |
| Healthy Control | SFC840-03-03 | Ctrl |
| (Lee et al., 2020) | SFC840-03-03 D11 | LRRK2-KO |
| LRRK2R1441C | JR207-03 | RC 1 |
| LRRK2R1441C | SFC869-03-04 | RC 2 |
| LRRK2R1441C | SFC870-03-05 | RC 3 |
LRRK2 R1441C (SFC869)
LRRK2 R1441C (SFC870)

## Slide 5
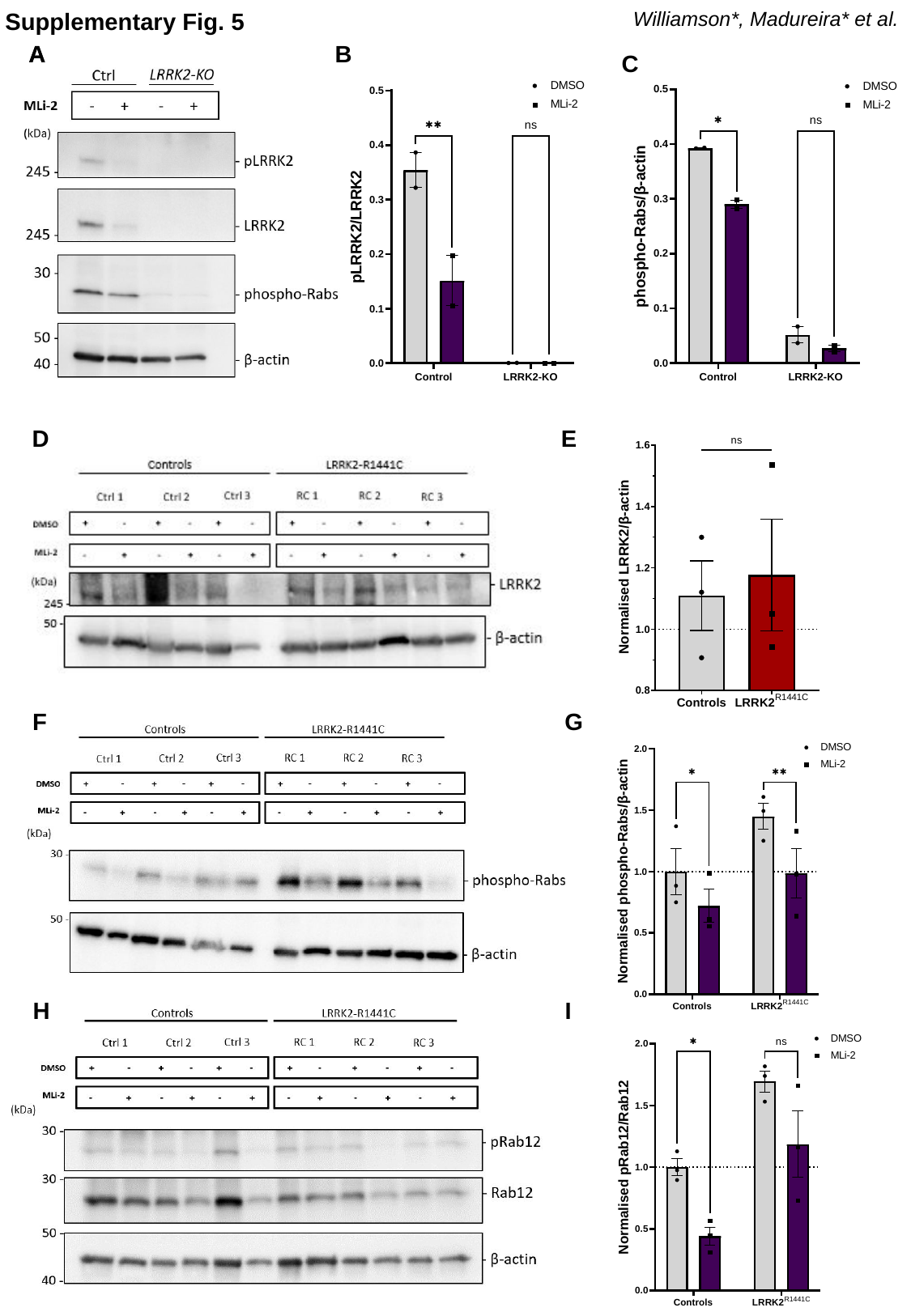

Williamson*, Madureira* et al.
Supplementary Fig. 5
A
B
A
C
D
E
G
F
I
H

## Slide 6
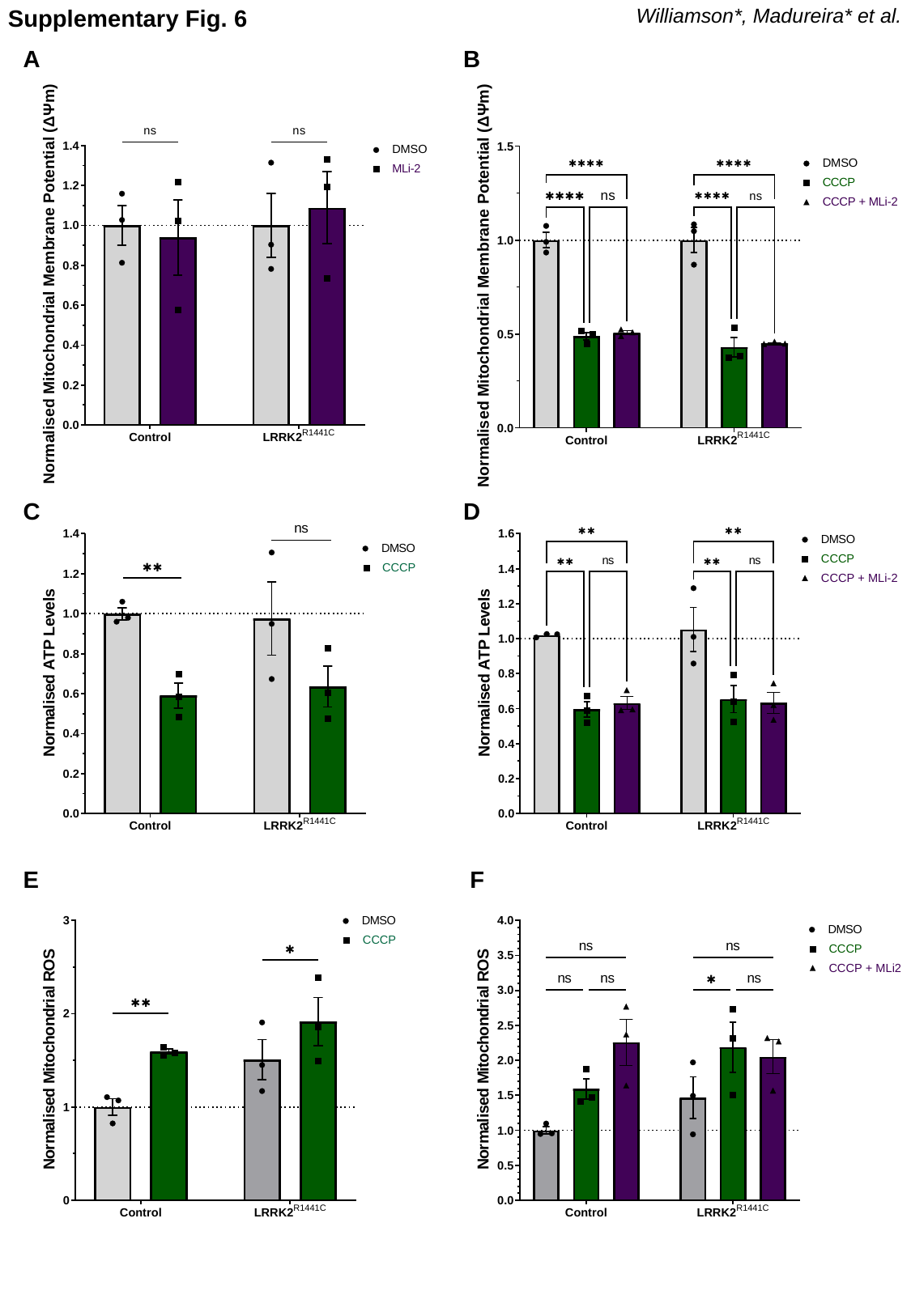

Williamson*, Madureira* et al.
Supplementary Fig. 6
A
B
D
C
E
F

## Slide 7
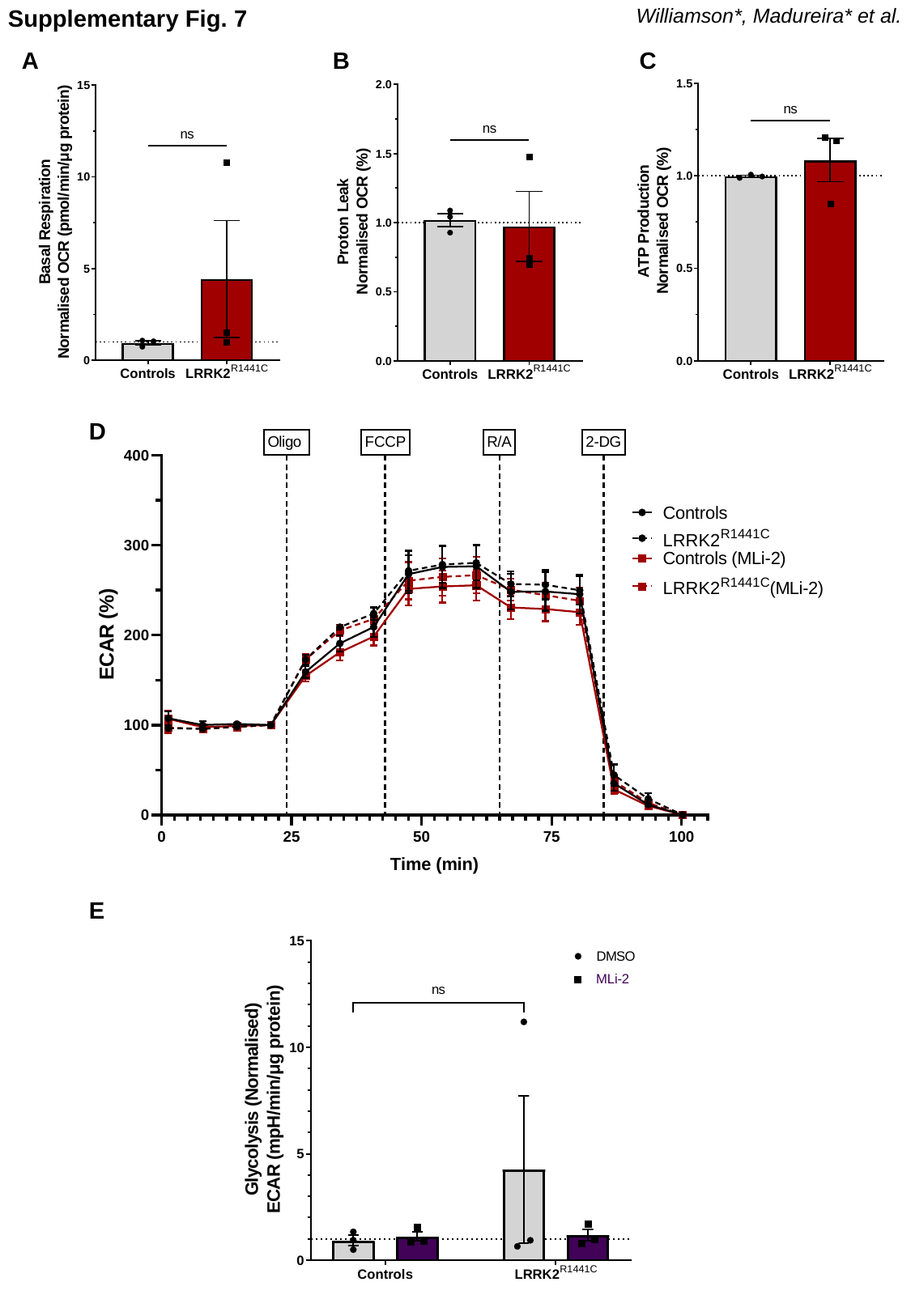

Williamson*, Madureira* et al.
Supplementary Fig. 7
A
B
C
D
E

## Slide 8
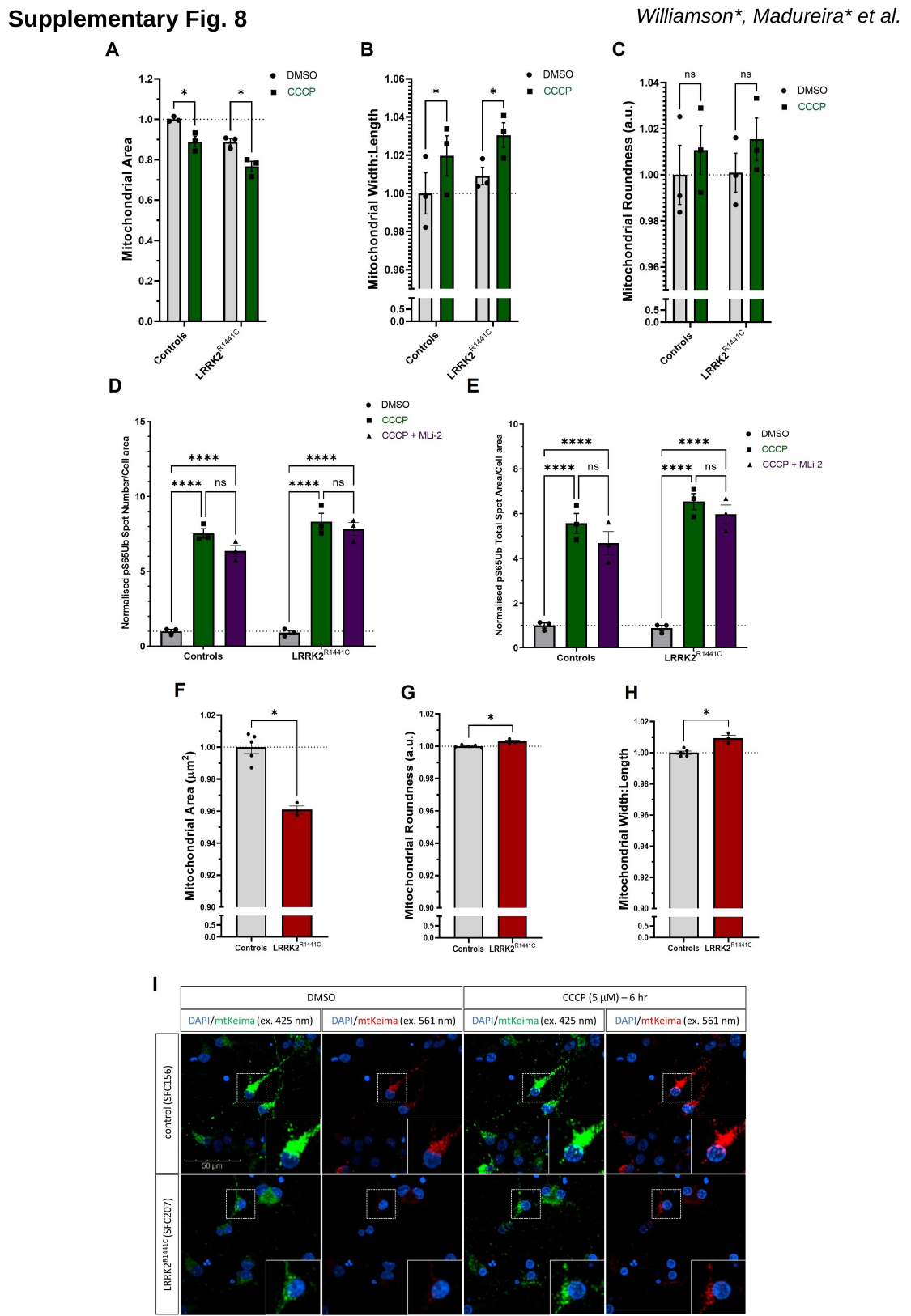

Williamson*, Madureira* et al.
Supplementary Fig. 8

## Slide 9
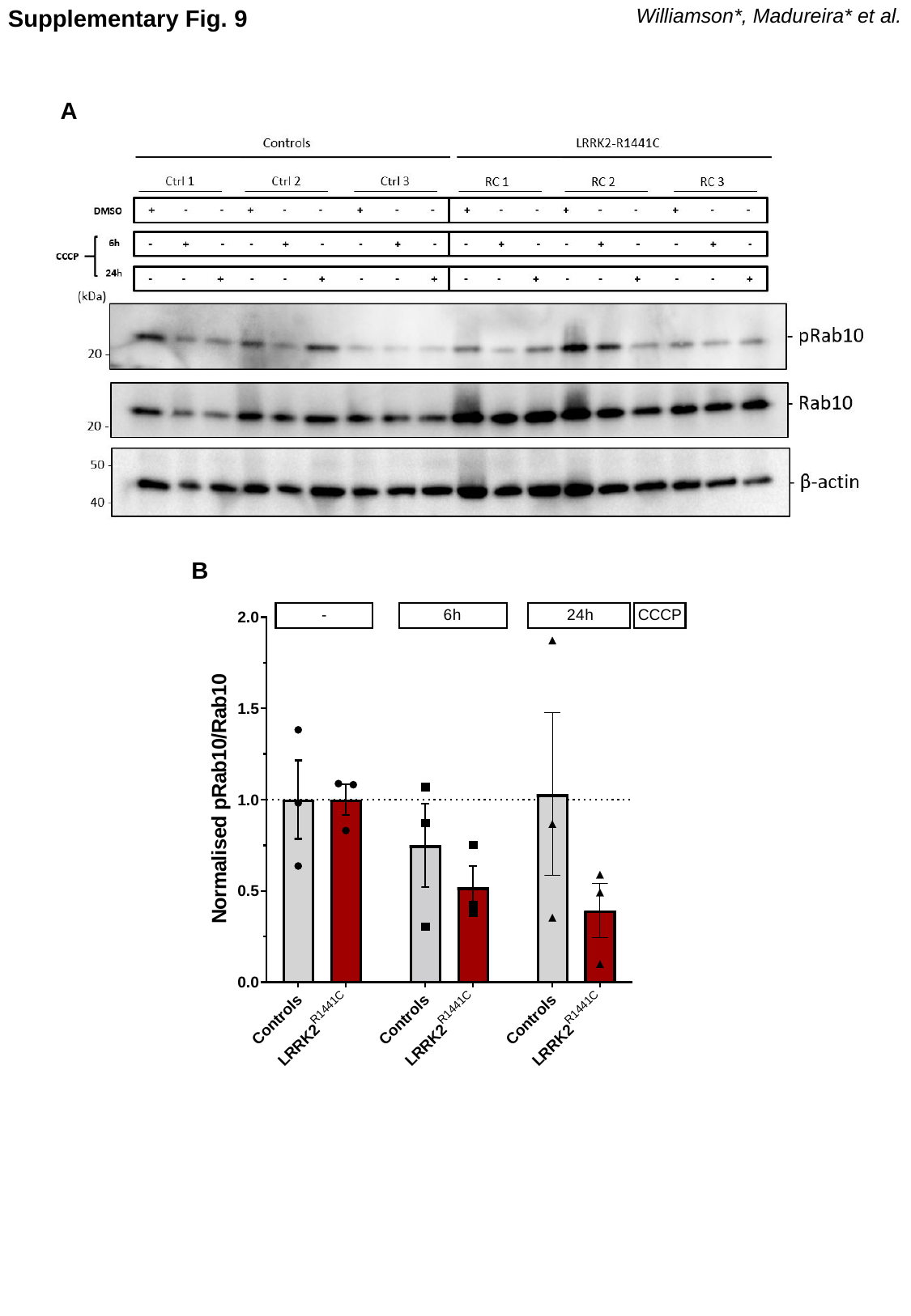

Williamson*, Madureira* et al.
Supplementary Fig. 9
A
B

## Slide 10
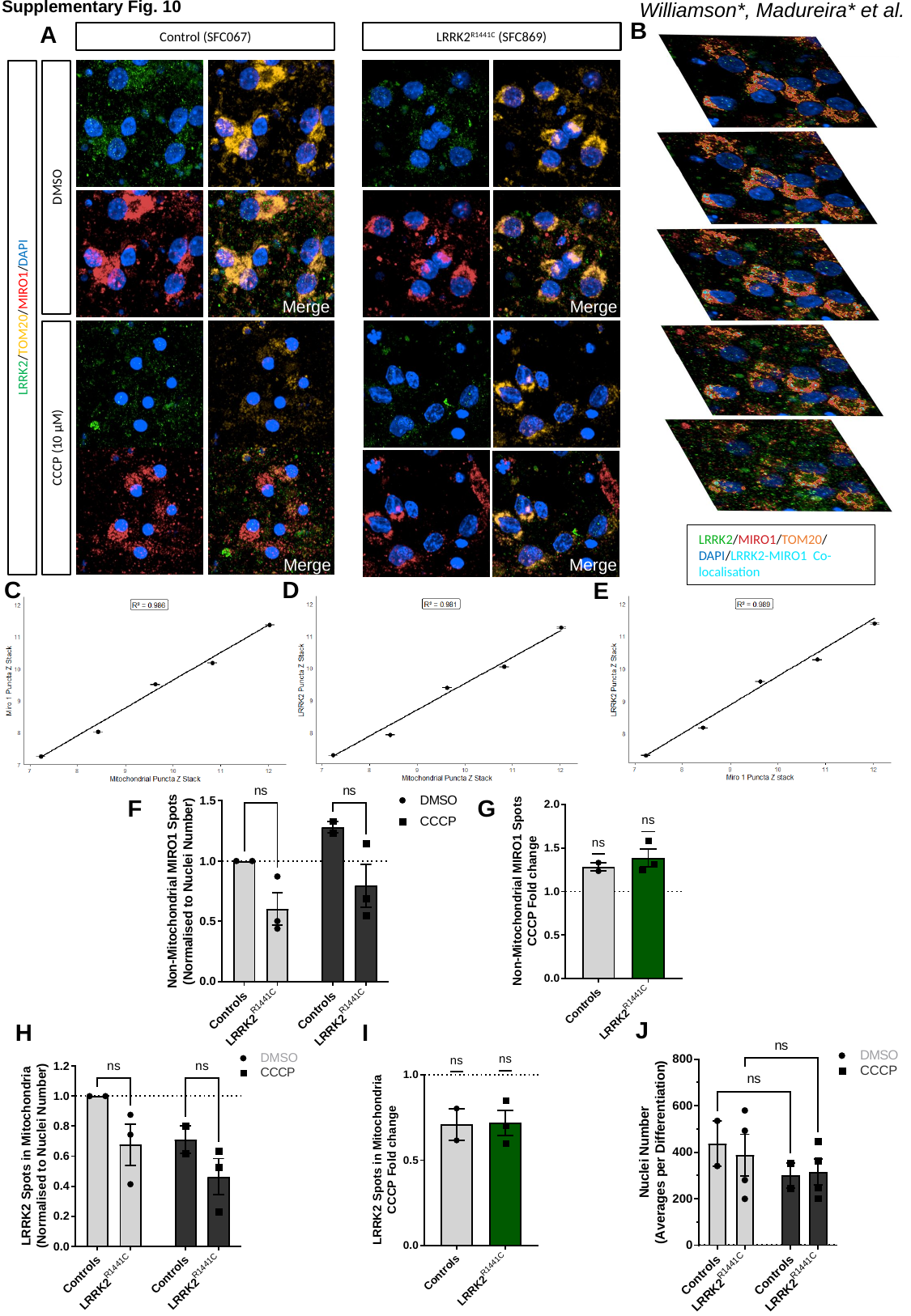

Supplementary Fig. 10
Williamson*, Madureira* et al.
B
A
Control (SFC067)
LRRK2R1441C (SFC869)
DMSO
Merge
Merge
LRRK2/TOM20/MIRO1/DAPI
CCCP (10 µM)
Merge
Merge
LRRK2/MIRO1/TOM20/DAPI/LRRK2-MIRO1 Co-localisation
C
D
E
F
G
J
H
I
